# Genomic analyses show extremely perilous conservation status of African and Asiatic cheetahs (*Acinonyx jubatus*)

**DOI:** 10.1111/mec.16577

**Published:** 2022-07-17

**Authors:** Stefan Prost, Ana Paula Machado, Julia Zumbroich, Lisa Preier, Sarita Mahtani‐Williams, Rene Meissner, Katerina Guschanski, Jaelle C. Brealey, Carlos Rodríguez Fernandes, Paul Vercammen, Luke T. B. Hunter, Alexei V. Abramov, Martin Plasil, Petr Horin, Lena Godsall‐Bottriell, Paul Bottriell, Desire Lee Dalton, Antoinette Kotze, Pamela Anna Burger

**Affiliations:** ^1^ Research Institute of Wildlife Ecology Vetmeduni Vienna Vienna Austria; ^2^ LOEWE‐Center for Translational Biodiversity Genomics, Senckenberg Museum Frankfurt Germany; ^3^ South African National Biodiversity Institute Pretoria South Africa; ^4^ Department of Ecology and Evolution University of Lausanne Lausanne Switzerland; ^5^ Institute for Ecology, Evolution and Diversity Goethe University Frankfurt Germany; ^6^ Animal Ecology, Department of Ecology and Genetics, Evolutionary Biology Centre, Science for Life Laboratory Uppsala Universitet Uppsala Sweden; ^7^ Institute of Evolutionary Biology, School of Biological Sciences University of Edinburgh Edinburgh UK; ^8^ Department of Natural History NTNU University Museum, Norwegian University of Science and Technology (NTNU) Trondheim Norway; ^9^ CE3C ‐ Centre for Ecology, Evolution and Environmental Changes & CHANGE ‐ Global Change and Sustainability Institute, Departamento de Biologia Animal, Faculdade de Ciências Universidade de Lisboa Lisbon Portugal; ^10^ Faculdade de Psicologia Universidade de Lisboa, Alameda da Universidade Lisbon Portugal; ^11^ Breeding Centre for Endangered Arabian Wildlife Sharjah United Arab Emirates; ^12^ Wildlife Conservation Society New York New York USA; ^13^ School of Life Sciences University of KwaZulu‐Natal Durban South Africa; ^14^ Zoological Institute Russian Academy of Sciences Saint Petersburg Russia; ^15^ Department of Animal Genetics University of Veterinary Sciences Brno Czech Republic; ^16^ Central European Institute of Technology University of Veterinary Sciences Brno (CEITEC Vetuni) Brno Czech Republic; ^17^ Rex Foundation Dunstable UK; ^18^ Genetics Department University of the Free State Bloemfontein South Africa

**Keywords:** *Acinonyx jubatus*, cheetah, conservation genomics, double‐digest restriction site associated DNA (ddRAD) sequencing, phylogeography

## Abstract

We live in a world characterized by biodiversity loss and global environmental change. The extinction of large carnivores can have ramifying effects on ecosystems like an uncontrolled increase in wild herbivores, which in turn can have knock‐on impacts on vegetation regeneration and communities. Cheetahs (*Acinonyx jubatus*) serve important ecosystem functions as apex predators; yet, they are quickly heading towards an uncertain future. Threatened by habitat loss, human‐wildlife conflict and illegal trafficking, there are only approximately 7100 individuals remaining in nature. We present the most comprehensive genome‐wide analysis of cheetah phylogeography and conservation genomics to date, assembling samples from nearly the entire current and past species' range. We show that their phylogeography is more complex than previously thought, and that East African cheetahs (*A. j. raineyi*) are genetically distinct from Southern African individuals (*A. j. jubatus*), warranting their recognition as a distinct subspecies. We found strong genetic differentiation between all classically recognized subspecies, thus refuting earlier findings that cheetahs show only little differentiation. The strongest differentiation was observed between the Asiatic and all the African subspecies. We detected high inbreeding in the *Critically Endangered* Iranian (*A. j. venaticus*) and North‐western (*A. j. hecki*) subspecies, and show that overall cheetahs, along with snow leopards, have the lowest genome‐wide heterozygosity of all the big cats. This further emphasizes the cheetah's perilous conservation status. Our results provide novel and important information on cheetah phylogeography that can support evidence‐based conservation policy decisions to help protect this species. This is especially relevant in light of ongoing and proposed translocations across subspecies boundaries, and the increasing threats of illegal trafficking.

## 1 INTRODUCTION

The current century is characterized by unprecedented global change. Climate change and human impacts disrupt the integrity of ecosystems worldwide with ramifications for faunal and floral biodiversity (Díaz et al., 2019; Eichenberg et al., 2021; Raven & Wagner, 2021). An estimated one million animal and plant species are threatened by extinction (Díaz et al., 2019). Apex predators, such as big cats, are more sensitive to environmental and climate change than species at lower trophic levels (Cheng et al., 2017; Voigt et al., 2003). Strong declines or extinctions of large carnivores can have substantial effects on ecosystems, such as an uncontrolled increase in wild herbivores, which in turn can alter plant communities and lead to shifts towards alternative ecosystem states (Beschta & Ripple, 2009) and even effect grassland net nitrogen mineralization (Frank, 2008).

The cheetah (*Acinonyx jubatus*, Schreber, 1775) is listed globally as a Vulnerable species by the International Union for Conservation of Nature (IUCN; Durant et al., 2015). However, as the majority of cheetahs occur outside formally protected areas, where rates of decline are likely to be elevated, Durant and colleagues argued that the cheetah meets the IUCN criteria to be categorized as *Endangered* (Durant et al., 2017). Furthermore, the subspecies *A. j. venaticus* in Iran and *A. j. hecki* in Northwest Africa are listed as *Critically Endangered* by the IUCN (Belbachir, 2008; Durant et al., 2015; Jowkar et al., 2008). Threats to cheetahs include habitat conversion and loss, persecution by pastoralists, prey declines, illegal trophy hunting, illegal trade especially as pets, and armed conflicts (Durant et al., 2014; Lindsey et al., 2011; Ray, 2005; Tricorache et al., 2018, 2021).

**FIGURE 1:**
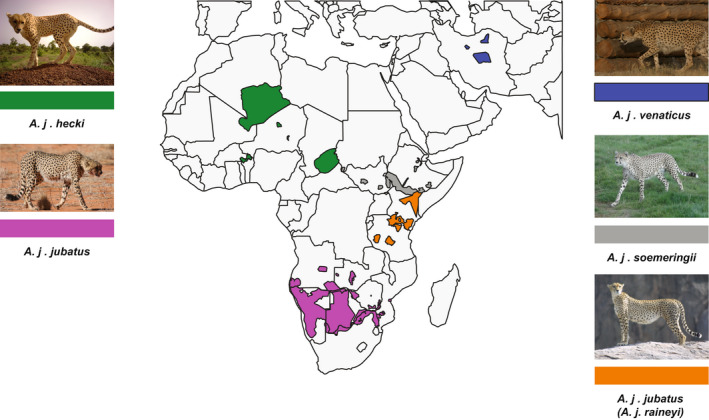
Current distribution of the five classical cheetah subspecies (after Krausman & Morales, 2005). The distribution ranges were adopted from the IUCN red list (Durant et al., 2015, 2017). Subspecies were assigned to the distributions using the results of Charruau et al. (2011) and this study. Photo credits are listed in the Acknowledgements.

There are approximately 7100 adult and adolescent cheetahs distributed across 33 wild subpopulations in Africa and Asia (Durant et al., 2017; Figure 1). More than half (~60%) of wild cheetahs occur in one large population in southern Africa (*A. j. jubatus*) (Durant et al., 2017), while *A. j. venaticus* is represented by fewer than 50–70 individuals and is only found in Iran (Farhadinia et al., 2017). At the end of the nineteenth century, the cheetah's distribution comprised most nonrainforest parts of Africa and much of Western and Southern Asia, from the Arabian Peninsula all the way to India, and northwards until Kazakhstan (Durant et al., 2017). However, over the past decades, the species' range declined drastically, and its current extent is probably only 9% of its historical distribution (Durant et al., 2017). Currently cheetahs are divided into four subspecies by the IUCN Cat Specialist Group (Kitchener et al., 2017), namely *A. jubatus hecki* (Northwest Africa), *A. j. soemmeringii* (Northeast Africa), *A. j. jubatus* (Southern and East Africa) and *A. j. venaticus* (Western and Southern Asia, presently found only in Iran). Krausman and Morales (2005) list *A. j. raineyi* (East Africa) as a fifth subspecies, but its status is under debate (Kitchener et al., 2017). It is currently recognized as a synonym of *A. j. jubatus*, because of its close genetic relationship inferred from mitochondrial DNA (mtDNA; Charruau et al., 2011), a finding that has previously been reported (O'Brien et al., 1987). Based on mtDNA and microsatellite data from up to 94 samples including most of the cheetah's past and current range, Charruau et al. (2011) further showed that Asiatic, North‐East and West African cheetahs form separate phylogenetic groups, corresponding to the currently recognized cheetah subspecies.

Recognizing the need for comprehensive conservation genomic analyses in cheetahs, we investigated genome‐wide single nucleotide polymorphisms (SNPs), mtDNA and major histocompatibility complex (MHC) class II DRB immune response gene data to examine their phylogeography, and neutral and adaptive genetic diversity. We used the classical subspecies assignment based on geographic distribution as a starting point (hypothesis) to test the validity of subspecies (Sackett et al., 2014): *A. j. hecki*, *A. j. soemmeringii*, *A. j. jubatus, A. j. venaticus* and *A. j. raineyi*, acknowledging that the latter is currently not recognized by the IUCN as a separate subspecies (Kitchener et al., 2017). Discussing this information in the context of ongoing and future conservation measures, we intend that our data build the basis for comprehensive range‐wide genetic monitoring of cheetahs. Moreover, it can be used to guide subspecies‐specific conservation measures, as it sheds light on the genetic differentiation among cheetah subspecies, and will assist with evidence‐based decision‐making, for example, for planned reintroduction projects and regional conservation strategies.

## 2 MATERIALS AND METHODS

A detailed description of the applied methods and all Convention on International Trade in Endangered Species of Wild Fauna and Flora (CITES) permits can be found in Supporting Information File 1 and Table S1. Information about all samples used in this study can be found in Table S2.

### 2.1 Data set descriptions

#### 2.1.1 Data set 1—nuclear DNA

Data set 1 consists of 46 *Acinonyx jubatus* individuals used in the population genomic analyses (belonging to all five classically recognized subspecies), and 12 *A. j. jubatus* individuals of known parent‐offspring trios only used for testing the reliability of the relatedness tests (see Table 1). Sequencing reads for three individuals in data set 1 were obtained from NCBI (Genbank: SRR2737543‐SRR2737545; Dobrynin et al., 2015). The data set also includes a specimen of *Puma concolor* used as outgroup in the phylogenetic analyses. We extracted DNA from tissue samples of 21 modern cheetahs and of the puma using the Qiagen DNeasy Blood and Tissue kit (Qiagen) and 32 diluted blood samples using the innuPREP Blood Kit (Analytik Jena AG). We extracted the DNA of the two museum samples of *A. j. hecki* using the DNA extraction protocol developed by Dabney et al. (2013). All but the two museum samples were processed using the double‐digest restriction‐site associated DNA (ddRAD) sequencing approach (Peterson et al., 2012). The two museum specimens were sequenced for their whole genomic DNA using the method described in Meyer and Kircher (2010), as their quality was not high enough to carry out ddRAD processing. To avoid DNA contamination, we carried out all extractions in a dedicated laboratory for ancient DNA and museum samples at the University of Uppsala, Sweden.

**TABLE 1:** Number of individuals included in the different analyses (third column) based on genome‐wide SNP data. The method used to generate the sequencing data is given in the right column

Subspecies	Number	Use	Method
*A. j. jubatus*	23	Population genomics	ddRAD
*A. j. soemmeringii*	15	Population genomics	ddRAD
*A. j. raineyi*	3	Population genomics	Dobrynin et al. (2015)
*A. j. venaticus*	3	Population genomics	ddRAD
*A. j. hecki*	2	Population genomics	Whole genomic DNA from museum samples
*A. j. jubatus*	12	Relatedness testing	ddRAD; known parent‐offspring trios
*Puma concolor*	1	Outgroup	ddRAD

##### Raw data processing

We mapped the read data against the Aci_jub_2 cheetah genome assembly (NCBI: GCA_003709585.1) using BWA‐MEM (version 0.7.17‐r1188) (Li & Durbin, 2010) and processed the resulting mapping files using Samtools (version 1.9) (Li et al., 2009). We subsequently assessed the mapping quality with Qualimap (Okonechnikov et al., 2016). For the two museum samples we further carried out adapter‐trimming and duplicate removal using AdapterRemoval2 (Schubert et al., 2016) and Picard version 2.8.2 (https://broadinstitute.github.io/picard/). The resulting mapping files were processed with ANGSD (Korneliussen et al., 2014), which was specifically developed for population genomic analyses of low coverage data. We carried out filtering using SNPcleaner (version 2.24) (Fumagalli et al., 2014), by first creating a vcf file (SNP data file) for all samples using Samtools. We then filtered the SNP data for (1) the presence of no more than 25% of missing data for individual sites, (2) a maximum coverage of 120x per individual to avoid calling sites in highly repetitive regions, and (3) a minimum coverage of 3x for each individual. The resulting sites were used as input for the downstream ANGSD analyses.

##### Genomic differentiation

The following analyses were carried out with the 46 individuals from data set 1 (excluding the 12 parent‐offspring individuals only used for the relatedness test assessment). We carried out unsupervised principal component analyses using ANGSD and pcangsd (Meisner & Albrechtsen, 2018) and investigated population differentiation in terms of *F*_ST_ with ANGSD. As some estimators of *F*_ST_ can be biased by uneven sampling (see e.g., Puechmaille et al., 2016), we created three replicates for which we randomly subsampled *A. j. jubatus* and *A. j. soemmeringii* down to three individuals. All replicates resulted in highly similar estimates (Table S3), comparable to the estimates obtained from the full data set (Table S4). Furthermore, Nazareno et al. (2017) and Willing et al. (2012) showed that *F*_ST_ can be reliably estimated based on small sample sizes (*n* > 2) when more than 1000–1500 SNPs are used in the analysis. To further investigate the reliability of our *F*_ST_ estimates, we carried out a permutation test. To do so, we generated three data sets, mimicking our original sampling (four groups of three individuals and one group of two individuals), in which each group was made up of randomly selected individuals of different subspecies. We then checked the *F*_ST_ distributions, the random ones and the one obtained from the original set for normality with the Shapiro–Wilk test in the R (version 3.4.3.) stats package. As both followed a normal distribution, we next tested whether both distributions had the same variance using the var.test() function of the stats package. This showed that both distributions are independent, and we thus proceeded with an F‐test (implemented in the stats package) to see whether both were significantly different from each other.

We then looked for signatures of admixture using ngsAdmix (Skotte et al., 2013). To avoid sampling biases due to different sample sizes, we subsampled *A. j. jubatus* and *A. j. soemmeringii* to three individuals to match the sample sizes of the other three subspecies. We generated three replicates with different subsampled individuals. We further carried out a separate ngsAdmix analysis restricted to all individuals of the two subspecies *A. j. soemmeringii* and *A. j. jubatus*. We performed 50 replicates for all ngsAdmix runs ranging from *k* = 2 to *k* = 5. The results were summarized and visualized using CLUMPACK (Kopelman et al., 2015). We assessed the model fit of each k value to the data using evalAdmix (Garcia‐Erill & Albrechtsen, 2020). Finally, we performed an EEMS (Petkova et al., 2016) analysis to infer effective migration rates between the African cheetah subspecies.

##### Phylogenetic analyses

We carried out phylogenetic analyses using three different methods: (1) using genetic distances, generated with ANGSD and ngsDist (Fumagalli et al., 2014), and FastME (Lefort et al., 2015), (2) a phylogenetic network approach using SplitsTree (Huson et al., 2008) and (3) a maximum likelihood based analysis with IQ‐Tree (Nguyen et al., 2015). Methods (1) and (2) were based on genotype likelihoods, while method (3) was based on genotype calls.

##### Conservation genomic parameters

We investigated classical conservation genomic parameters, such as inbreeding, relatedness and heterozygosity. Inbreeding was estimated using two approaches: (1) using ngsF (Fumagalli et al., 2014), and (2) ngsRelate (version 2) (Hanghøj et al., 2019). We further used ngsRelate version 1 (Korneliussen & Moltke, 2015) and version 2 (Hanghøj et al., 2019), the latter corrects for inbreeding, to carry out relatedness analyses. To check test performance, we first ran the relatedness analysis tools on only the 12 individuals consisting of four parent‐offspring trios (IDs: 448–459; see Table S2).

We carried out heterozygosity analyses for the 46 individuals using realSFS (part of the ANGSD package). Here, we did not restrict our analyses to filtered sites, to be able to compare our estimates to published genome‐wide heterozygosity values for other felids (Pečnerová et al., 2021). Due to the low coverage and quality typical for degraded museum DNA, we did not include the two museum samples in this analysis.

##### 2.1.1.1 Data set 2—mitochondrial DNA

We amplified mtDNA regions of 929 bp for 57 individuals and 681 bp for 57 individuals. We targeted two mitochondrial genes that included the 14 previously described diagnostic SNPs from Charruau et al. (2011), the NADH‐dehydrogenase subunit 5 (MT‐ND5) and the control region (MT‐CR). The amplicons were Sanger sequenced. We further included the 78 individuals from Charruau et al. (2011) in the 681 bp data set. All individuals of the 929 bp data set are included in the 681 bp set, as the 929 bp fragment includes the shorter 681 bp fragment. The mtDNA data were used to infer population structure, and included samples from their present and past distributions. We followed the protocol of Rohland et al. (2010) for the DNA extraction from museum samples. To avoid DNA contamination, we carried out all extractions in a dedicated laboratory for museum samples at the Vetmeduni in Vienna, Austria.

##### Population structure

For the mitochondrial DNA data we aligned all sequences using Codon Code Aligner version 3.0.2 (Codon Code Corporation). We obtained the reference sequence for the cheetah mitochondrial genome from GenBank (accession no. NC_005212.1; Burger et al., 2004). We carried out parallel analyses on the two concatenated data sets that differed in the length of the region and the number of individuals (Table S2). The first analysis comprised the larger mtDNA fragment of 929 bp amplified in 58 individuals. The second analysis was based on the shorter fragment of 681 bp and included 78 individuals from Charruau et al. (2011) and 57 examined in this study. Median‐joining networks were created using Popart (Leigh & Bryant, 2015). We further investigated the molecular variance in our data using an AMOVA analysis, implemented in the software Arlequin version 3.5.2.2 (Excoffier & Lischer, 2010). Furthermore, we investigated the homology of the mtDNA regions to known NUMTs in the published cheetah genome (GCA_003709585.1) using the default settings in blastn (Altschul et al., 1990).

#### 2.1.2 Data set 3—mini‐barcodes

We designed a mini‐barcode approach to investigate whether all *A. j. soemmeringii* carry the 3 bp deletion in the MT‐ND5 gene as described in Charruau et al. (2011), and as a quick subspecies assignment test. We designed three mini‐amplicons that amplify a total of 190 bp, based on diagnostic sites inferred from our mitochondrial haplotype data (data set 2). Primer sequences can be found in Table S5. We used the extracts of all *A. j. soemmeringii* specimens from data set 1 and sequenced the amplicons on an Illumina iSeq 100 (2 × 150 bp) following the two step sequencing protocol of Lange et al. (2014).

##### Testing for the presence of the 3 bp deletion in the MT‐ND5 gene in *A. j. Soemmeringii*

We mapped all sequencing reads back to the mitochondrial DNA reference for cheetahs (accession no. NC_005212.1; Burger et al., 2004) using the same approach as described for data set 1. We then called the consensus sequences of the mapping files using ANGSD (option: ‐do Fasta 3), inspected them by eye, and aligned all consensus sequences using Mafft (Katoh & Standley, 2013). Then we generated median joining networks for the mini‐barcode data using Popart (Leigh & Bryant, 2015).

#### 2.1.3 Data set 4—immune response genes

We sequenced the MHC class II DRB exon 2 of 46 individuals (belonging to four of the five classical subspecies; excluding *A. j. hecki*) to investigate their immunogenetic diversity. We used the Qiagen DNeasy Blood and Tissue kit for DNA extraction from hair and tissue samples, and the VWR PeqGold Tissue DNA Mini Kit Plus for blood samples. We carried out PCR amplifications of the target region as described in Castro‐Prieto, Wachter and Sommer (2011). Indexing, multiplexing and sequencing was carried out following the Illumina Nextera XT DNA Library Prep kit reference guide. Sequencing was performed on an Illumina MiSeq (2 × 250 bp).

##### Adaptive immune system diversity

We mapped the reads against the MHC class II DRB exon 2 reference sequence from Castro‐Prieto, Wachter and Sommer (2011) using BWA‐MEM, and further processed the mapping file using Samtools. We called variants using Picard version 2.8.2 and GATK version 3.1.8 (McKenna et al., 2010). The main alleles were phased using the FastaAlternateReferenceMaker command in GATK. We estimated haplotype diversity (Hd) and nucleotide diversity (π) using DNAsp version 6.12.03 (Librado & Rozas, 2009). Rarefaction analyses were carried out using EstimateS 9.1.0 (Colwell & Elsensohn, 2014).

## 3 RESULTS

For the ddRAD data we aimed for a minimum of 225,000 read pairs per individual, assuming the generation of 45,000 loci. We obtained 44,351 loci, and read pair counts ranged from 120,700 to 38.9 million for the different individuals, with an average read pair count of 3.58 million. For the two museum samples we aimed at 125 million read pairs and obtained 127.6 and 176 million read pairs, respectively. After filtering for coverage and missing data we retained 3743 SNPs for the analyses.

### 3.1 Genomic differentiation among the five classical cheetah subspecies

**FIGURE 2:**
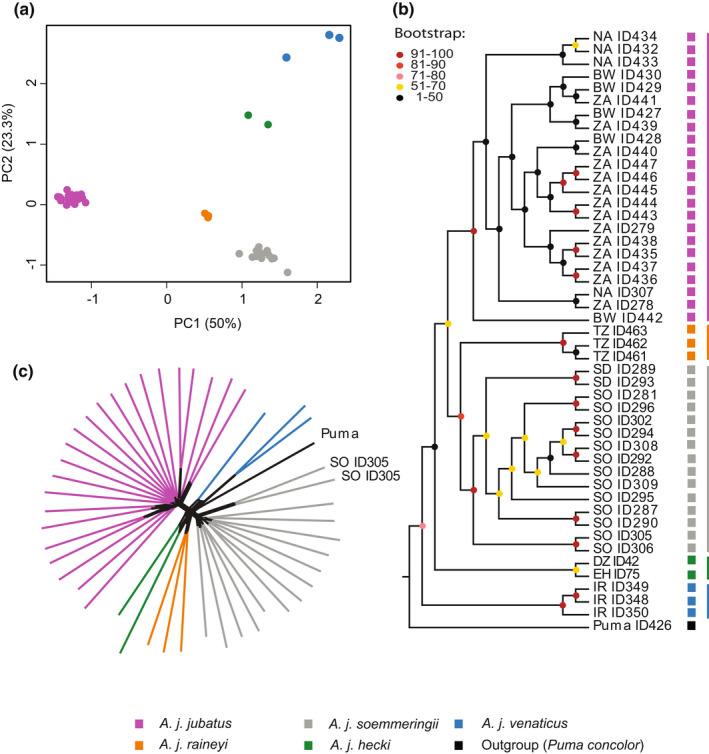
Population‐ and phylogenomic analyses of genome‐wide nuclear SNP (3743) data for 46 cheetah individuals. (a) PCA analysis of all individuals of the five classical subspecies included in this study. The clustering corresponds to the morphological subspecies classification. (b) Phylogenetic relationships of representatives of the five classical cheetah subspecies using genetic distances (estimated using ngsDist and FastME; using 100 bootstraps). (c) Phylogenetic relationships of representatives for the five classical cheetah subspecies inferred by the phylogenetic network approach implemented in SplitsTree. For a fully annotated phylogenetic network see Figure S3.

**FIGURE 3:**
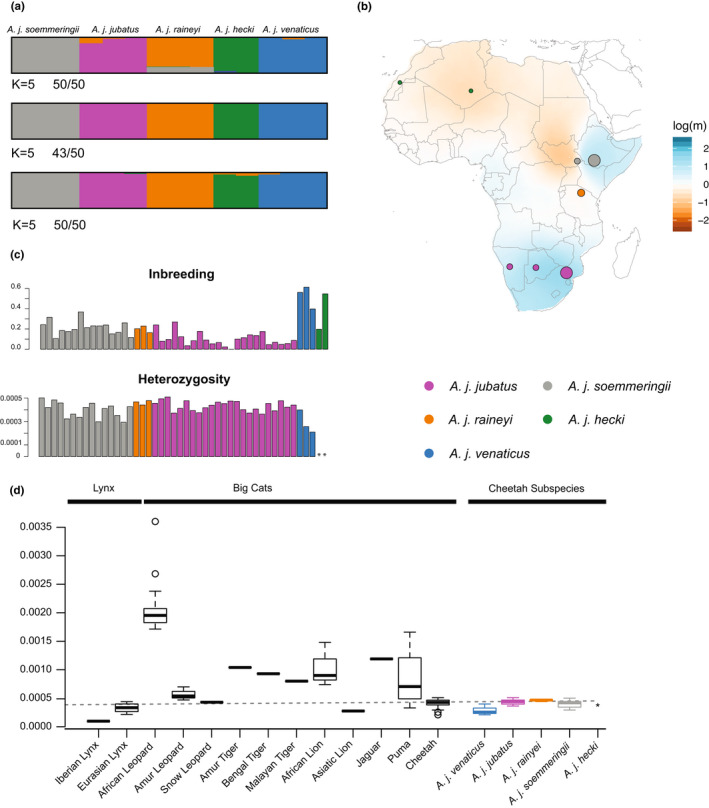
Population genetic analyses of cheetahs using genome‐wide SNP data. **(**a) Admixture analyses for K = 5 for the three different sample subsets using the 3743 SNP data. Numbers indicate how many individual runs of the 50 replicates support this grouping. (b) Effective migration rates between the African cheetah subspecies. Blue and brown colours reflect below‐ and above‐the‐average migration rates, respectively. The dots represent approximate sample locations (exact locations were not available) and their size is proportional to the number of samples from this region. (c) Inbreeding coefficients based on the 3743 SNPs and heterozygosity based on genome‐wide data indicating high inbreeding in individuals of *A. j. Venaticus* and *A. j. hecki* and low heterozygosity in individuals of *A. j. Venaticus*. **Indicates that individuals of *A. j. Hecki* were not included in the heterozygosity analysis. (d) Genome‐wide heterozygosity data for big cats, two lynx species, and five cheetah subspecies. All values apart from the cheetah estimates were obtained from Pečnerová et al. (2021). *Indicates that individuals of *A. j. Hecki* were not included in the heterozygosity analysis. The dashed line indicates the average value for the cheetah as a species.

Subspecies or conservation unit assignments are crucial to carry out targeted conservation efforts. Therefore, we analysed biogeographic relationships using genome‐wide SNP data (3743 SNPs after filtering) for 46 individuals of the five classically recognized cheetah subspecies (see Table 1 and Table S2; data set 1). In contrast to the current and in line with the classical subspecies taxonomy, the unsupervised principal component analysis (PCA) showed five distinct genomic clusters (Figure 2a and Figure S1). These clusters correspond to the four currently recognized subspecies (*A. j. jubatus*, *A. j. soemmeringii*, *A. j. hecki* and *A. j. venaticus*) and *A. j. raineyi*. This is further supported by the two phylogenetic tree analyses (Figure 2b, Figure S2) and the phylogenetic network approach (Figure 2c, Figure S3). These analyses show monophyletic groups for the five subspecies (note that we could not include the two *A. j. hecki* samples in the ML tree analysis, which is based on genotype calls, due to their low coverage). The two phylogenetic tree analyses showed high support for these clades, with bootstraps falling in the range of 91%–100%, except for the *A. j. hecki* clade, which shows a bootstrap of 70% (Figure 2b and Figure S2). Overall, the two phylogenetic tree topologies were congruent with each other and placed *A. j. venaticus* (from Asia) as a sister group to all African subspecies. However, they differed in the placement of two individuals (ID 305 and ID306), which appeared as sisters to (i) *A. j. soemmeringii* using genetic distances (Figure 2b), or (ii) *A. j. raineyi* in the ML tree (Figure S2). These individuals showed mitochondrial haplotypes associated with *A. j. raineyi* (see below), but clustered with *A. j. soemmeringii* in the PCA analysis based on the genome‐wide SNP data. The split into five genetically distinct groups is further supported by model fit analyses based on the inferred admixture proportions (Figure 3a and Figures S4 and S5). Analyses of different subsets (at *K* = 5) indicated little to no signatures of admixture (Figure 3a and Figure S4); one of the three replicate analyses suggested some, but limited, *A. j. soemmeringii* ancestry in *A. j. raineyi* individuals and, likewise, some slight *A. j. raineyi* ancestry in *A. j. jubatus* individuals, but no signatures or patterns of admixture were consistently observed across the three different sample subsets analysed. We ran a separate admixture analysis including all individuals of *A. j. soemmeringii* and *A. j. jubatus*, but did not detect any signatures of admixture between these two subspecies (Figure S6). To understand patterns of gene flow among African cheetah populations, we estimated effective migration rates using EEMS (Figure 3b). This analysis indicated (1) migration between populations of the same subspecies (except for the two *A. j. hecki* individuals), (2) limited to no migration between *A. j. jubatus* and *A. j. raineyi*, and *A. j. soemmeringii* and *A. j. raineyi*, and (3) strong migration barriers between *A. j. hecki* and all the other African subspecies. Genetic distances measured using *F*_ST_ were the highest (0.497) between the two endangered subspecies, *A. j. hecki* and *A. j. venaticus*, and lowest (0.219) between *A. j. soemmeringii* and *A. j. raineyi* (Table 2). In general, we saw the highest *F*_ST_ values between *A. j. venaticus* and all the African subspecies (ranging from 0.438 to 0.497). The permutation test showed that our *F*_ST_ were reliable, with a *p*‐value of .0003354. The *F*_ST_ estimates for the replicates of the subsampled data sets and the complete data set can be found in Tables S3 and S4.

**TABLE 2:** *F*_ST_ values between the five classical subspecies of cheetahs. For this analysis we downsampled *A. j. Soemmeringii* and *A. j. jubatus* to three individuals each

*FST*	*A. j. jubatus* (*n* = 3)	*A. j. venaticus* (*n* = 3)	*A. j. hecki* (*n* = 2)	*A. j. raineyi* (*n* = 3)
*A. j. soemmeringii* (*n* = 3)	0.285	0.438	0.384	0.219
*A. j. jubatus* (*n* = 3)		0.471	0.384	0.247
*A. j. venaticus* (*n* = 3)			0.497	0.475
*A. j. hecki* (*n* = 2)				0.369

### 3.2 High inbreeding and low heterozygosity threaten the gene pool of the critically endangered Asiatic and northwest African cheetahs

We carried out inbreeding analyses using data set 1 and two different methods described in Fumagalli et al. (2014) and Hanghøj et al. (2019). Both inferred the highest inbreeding coefficients in individuals of the two *Critically Endangered* subspecies, *A. j. venaticus* and *A. j. hecki* (Figure 3c top panel and Figure S7), although with slightly different intensities. *A. j. jubatus* showed slightly lower inbreeding coefficients than *A. j. soemmeringii* or *A. j. raineyi* (Figure 3c top panel and Figure S7). While most of the *A. j. jubatus* individuals are captive bred (from South African captive breeding facilities), we did not observe any differences between these and the wild *A. j. jubatus* individuals in our sampling. We further calculated genome‐wide heterozygosity for each of the modern samples (excluding the low‐quality museum samples of *A. j. hecki*), which resulted in a species mean of 0.00040 (range: 0.00020–0.00050; Figure 3c bottom panel and Figure 3d). *A. j. venaticus* showed the lowest genome‐wide heterozygosity with a mean of 0.00029 (range: 0.00020–0.00040), followed by *A. j. soemmeringii* with a mean of 0.00040 (range: 0.00029–0.00050), *A. j. jubatus* with a mean of 0.00043 (range: 0.00036–0.00050) and *A. j. raineyi* with a mean of 0.00046 (range: 0.00044–0.00048). In addition, we performed relatedness analyses to assess the impact of relatedness on our analyses. Most *A. j. jubatus* and *A. j. soemmeringii* showed relatedness patterns (coefficient of relatedness [*r* = *k*2/2 + *K*1] and *k*0) indicative of second to fourth generation cousins (Figure S8). A very limited number of individuals showed sibling or parent‐offspring relationships. Both methods were able to resolve relatedness of four parent‐offspring trios included in the data set (Figure S9). Comparisons between relatedness values (*k*2) using both methods can be found in Figure S10.

### 3.3 Population structure using mitochondrial DNA

**FIGURE 4:**
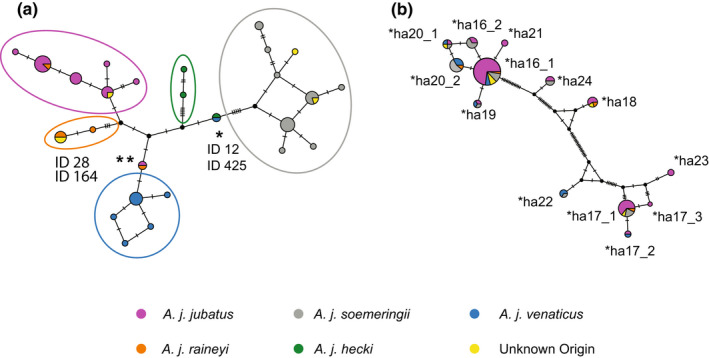
Haplotype analyses of (a) mitochondrial DNA data, and (b) MHC. II DRB exon 2 data. (a) Median‐joining haplotype network reconstruction based on 929 bp of mitochondrial DNA from 58 individuals. We circled the nominal haplogroups in the colours to which subspecies they can be assigned to. We marked the placement of the individual from India (ID 425) and the individual from Chad (ID 12) with an asterisk, and the individual from Tanzania (ID28) and the individual from Zimbabwe (ID164) with two asterisks. (b) Median‐joining network of the nucleotide sequences of the MHC II DRB exon 2.

The analyses of mtDNA fragments of different sizes allowed us to investigate population genetic structure throughout most of the cheetahs' present and historical range (Figure 4a; data set 2 and 3). We analysed mtDNA fragments of 681 bp from 135 individuals (Figure S11; data set 2), 929 bp from 58 individuals (Figure 4a; data set 2), and 190 bp of the 12 *A. j. soemmeringii* individuals for which we obtained nuclear SNP data (using a mini‐barcode approach; Figure S12; data set 3). The median‐joining networks showed distinct haplogroups for the five classical subspecies (Figure 4a, Figure S13), although with limited genetic variation differentiating them. However, we found that individuals of *A. j. raineyi* fell into two distinct mtDNA haplogroups. Individuals of *A. j. raineyi* fell either within the *A. j. jubatus* haplogroup (circled in purple in Figure 4a) or in a separate cluster, here referred to as the *A. j. raineyi* haplogroup (circled in orange in Figure 4a). As noted in Charruau et al. (2011), we found all *A. j. soemmeringii* mitochondrial haplotypes to display the 3 bp deletion in the MT‐ND5 gene. However, two out of the 12 *A. j. soemmeringii* individuals (data set 1), which clearly fell within the *A. j. soemmeringii* cluster based on nuclear SNP data (see Figure 2a), carried a mitochondrial haplotype belonging to the *A.j. raineyi* mtDNA haplogroup (Figure S12). We found a large fraction of the total variation partitioned between the five subspecies in the hierarchical AMOVA analysis using the mtDNA data (85% between subspecies and 15% within populations).

Over all our geographical sampling we discovered four unexpected haplotypes considering their sampling locations: we found an individual from Tanzania (ID 28) and one from Zimbabwe (ID 164) with similar haplotypes to *A. j. venaticus* individuals (one mutation difference; Figure 4a, Figure S11). We cannot rule out that these samples were mislabelled sometime after their field collection. Also, we did not find any significant homology of these sequences to nuclear mitochondrial DNA (NUMTs) copies in the published cheetah genome (NCBI: GCA_003709585.1). Furthermore, one of the two Indian individuals included in our study (ID 425, see Table S2) shared a haplotype with an individual from Chad (ID 12, *A. j. hecki*, Northwest Africa; Figure 4a). This could be due to the well‐documented historic cheetah trade from Africa into India (Divyabhanusinh, 1999). Interestingly, this haplotype (carried by ID 425 and ID 12) fell between the *A. j. hecki* and the *A. j. soemmeringii* haplogroup. Based on the location we would expect the Chad sample to cluster with the *A. j. hecki* haplogroup. More data, such as complete mitochondrial genomes or genome‐wide SNP data will be necessary to determine whether these are real signals, mislabeling, or artefacts caused by the short length of the analysed mtDNA fragments.

### 3.4 Adaptive immune response gene diversity

We sequenced the MHC class II DRB exon 2 of 46 individuals (belonging to four of the five classical subspecies—all but *A. j. hecki*; data set 4), which resulted in 13 nucleotide and nine amino acid (AA) haplotypes (Figure 4b, Table S6). We estimated a nucleotide haplotype diversity of 0.834 (standard deviation [std]: 0.028), a nucleotide diversity (π) of 0.069 (std: 0.005), and an average of 16.2 nucleotide differences (k). The most common AA haplotype was AcjuFLA‐DRB *ha16 carried by 83% of the individuals, followed by AcjuFLA‐DRB *ha17 and AcjuFLA‐DRB *ha20, found in 30% and 28% of the individuals, respectively (Table S6). Of the nine identified AA haplotypes, all were found in *A. j. jubatus*, seven in *A. j. soemmeringii* and five in *A. j. venaticus* (Table S6). AcjuFLA‐DRB *ha21 and AcjuFLA‐DRB *ha23 were only present in *A. j. jubatus*. Similar to Drake et al., 2004, we found up to four different haplotypes in a single individual (Table S6). We further investigated whether we could expect to retrieve more haplotypes with increasing sample sizes using extrapolation (Colwell & Elsensohn, 2014; Figure S13). Even though confidence intervals were wide, especially for *A. j. venaticus* and *A. j. soemmeringii*, some trends were apparent: (1) the steady increase of haplotypes for *A. j. jubatus* indicates that we have probably not saturated the sampling of MHC class II DRB exon 2 haplotypes for this subspecies; (2) the rarefaction curve of *A. j. venaticus* reaches a plateau at about 15 samples, which would indicate that no more than seven haplotypes are expected for this subspecies; and (3) we might be able to find more haplotypes for *A. j. soemmeringii* with increased number of samples (Figure S13).

## 4 DISCUSSION

### 4.1 Genomic analyses support the classical distinction of five cheetah subspecies

The understanding of subspecies structure in cheetahs has important implications for their conservation as these are often used to assign conservation units. Here, we show that our genetic results support the previously established distinction of five cheetah subspecies by Krausman and Morales (2005): *A. j. jubatus*, *A. j. soemmeringii*, *A. j. venaticus*, *A. j. hecki* and *A. j. raineyi*. Our reasoning follows the criteria outlined in Sackett et al. (2014). (1) *Genotypic separation of putative subspecies*: We found five distinct clusters in the unsupervised PCA analysis (based on nuclear SNP data), which fit the five classically recognized subspecies. Although differentiation between cheetah subspecies has previously been suggested to be low based on mtDNA data (O'Brien et al., 2017), the genome‐wide *F*_ST_ values of the present study show a high differentiation between the subspecies. These findings are consistent with the results reported by Charruau et al. (2011) for 18 microsatellite loci. However, that study only included microsatellite data for three subspecies: *A. j. venaticus*, *A. j. soemmeringii* and *A. j. jubatus*. We show that genome‐wide *F*_ST_ values (0.219–0.497) are comparable or higher than those of other large felids, such as tigers (0.11–0.43, Liu et al., 2018), lions (0.16–0.28, Smitz et al., 2018) or African leopards (0.05–0.15, Pečnerová et al., 2021). Contrary to the mitochondrial data, *A. j. raineyi* displayed a closer relationship to *A. j. soemmeringii* than to *A. j. jubatus*, with which it was recently subsumed, both in the PCA and the *F*_ST_ analysis using the genome‐wide data (data set 1). Admixture and effective migration rate analyses indicated no or limited gene flow between the subspecies. We found two individuals assigned to *A. j. soemmeringii*, using genome‐wide nuclear SNP data, carrying mitochondrial haplotypes of the *A. j. raineyi* haplogroup (Figure S11). Interestingly, these two individuals did not show any signatures of recent admixture (Figure S14), which could hint towards the possibility that this pattern is caused by incomplete lineage sorting rather than admixture. More individuals are needed to formally test these different hypotheses. (2) *Spatial segregation*: For the most part, cheetah subspecies show strong geographic segregation, with the distributions of only *A. j. raineyi* and *A. j. soemmeringii* perhaps being parapatric (Figure 1). (3) *Monophyly in the phylogenetic tree reconstructions*: The five putative subspecies show monophyly in all phylogenetic tree reconstructions based on the nuclear DNA data (Figure 2b,c and Figure S2). For the most part this pattern is also observed in the haplotype network reconstruction using the mitochondrial DNA data (Figure 4a; see the “Population structure using mitochondrial DNA” section of the Results for exceptions). (iv) Relatively large and significant fraction of the total variation partitioned between the subspecies: We found a large fraction of the total variation partitioned between the five subspecies in the hierarchical AMOVA analyses using the mitochondrial DNA data (data set 2; 85% between subspecies and 15% within population). We were not able to calculate hierarchical AMOVA for the genome‐wide SNP data, as there are currently no available tools that can calculate this for low‐coverage data.

### 4.2 Phylogeography, conservation genomics and their implications

The geographic distribution of the four African cheetah subspecies is similar to that of other savannah‐dwelling large African mammals. African cheetahs are made up of Western, Northeastern, Eastern and Southern African groups. This pattern is, for example, similar to the distribution of the four currently recognized giraffe lineages (Winter et al., 2018; Coimbra et al., 2021), with a few regional differences. While the distributions of *A. j. soemmeringii* and *A. j. raineyi* meet at the border between Ethiopia, South Sudan, and Kenya, the distributions of *Giraffa reticulata* and *G. tippelskirchi* meet further south in Central Kenya. Similar to cheetahs, Southern African lions show a closer phylogenetic relationship with those in Eastern Africa than with those in Western Africa (Bertola et al., 2016). Furthermore, population genomic analyses for lions also indicate the presence of more than one genetic cluster in Tanzania (three for lions, and two in the case of the cheetah mitochondrial analysis). In contrast to cheetahs, where the Asian subspecies *A. j. venaticus* is sister to all African cheetah subspecies (Figure 2b and Figure S2), Asian lions cluster phylogenetically with North African lions (Bertola et al., 2016; de Manuel et al., 2020). Estimates of the divergence dates within cheetahs based on mitochondrial DNA or genome data vary strongly between analyses and range from 4.5 to 139 kya (see e.g., Charruau et al., 2011; Dobrynin et al., 2015; O'Brien et al., 2017; Rai et al., 2020). Also, confidence intervals in these studies often show a high degree of overlap (Rai et al., 2020) and the underlying phylogenetic trees often show limited bootstrap support for the splits separating the monophyletic subspecies clades (Charruau et al., 2011), making it difficult to decide on the sequence of subspecies divergences. Given the lack of a reliable average mutation rate estimate for cheetah nuclear DNA, the limited bootstrap support for the underlying topology even for the genome‐wide SNP data, and taking the lower coverage for some of our samples into consideration, we refrained from estimating divergence times, acknowledging the limitations of our data set. High‐coverage genome data from all five classically accepted subspecies will be needed to better understand the cheetah's phylogenetic history.

We found the highest levels of inbreeding in individuals of the two *Critically Endangered* subspecies, *A. j. hecki* and *A. j. venaticus*, further emphasizing their extremely perilous conservation status. Moreover, *A. j. venaticus* also showed very low genome‐wide heterozygosity. We excluded the *A. j. hecki* data from this analysis, because of the low DNA quality obtained from museum samples. Of all the subspecies, *A. j. jubatus* showed the lowest level of inbreeding. Genome‐wide heterozygosity was the highest in *A. j. jubatus* and *A. j. raineyi*. This is not surprising as *A. j. jubatus* makes up the largest continuous population of all cheetah subspecies and *A. j. raineyi* the second largest (Durant et al., 2017). We have to caution that a large fraction of our *A. j. jubatus* individuals (18 out of 25) originated in captivity. However, these represent purebred *A. j. jubatus*, and we did not observe any differences in the genome‐wide heterozygosity or inbreeding estimates between our captive‐bred and wild caught individuals. Our genomic analyses show that cheetahs have genome‐wide heterozygosity values (0.0002–0.0005) lower than those of other endangered mid‐size or large felids (data from Pečnerová et al., 2021; also noted in Dobrynin et al., 2015), such as African leopards (*Panthera pardus pardus*, classified as *Vulnerable*, 0.0017–0.0036), African lions (*Panthera leo leo*, classified as *Vulnerable*, 0.0007–0.0015), jaguars (*Panthera onca*, classified as *Near Threatened*, 0.00119) and tigers (*Panthera tigris*, classified as *Vulnerable*, 0.0008–0.0010), but similar to Snow leopards (*Panthera uncia*, classified as *Vulnerable*, 0.0004) and Asiatic lions (*Panthera leo persica*, classified as *Endangered*, 0.000276; comparable to *A. j. venaticus*), and higher than the Iberian lynx (*Lynx pardinus*, classified as *Endangered*, 0.0001). This highlights the perilous conservation status of the cheetah, especially that of the critically endangered subspecies *A. j. venaticus* and *A. j. hecki*.

### 4.3 Global change and cheetah conservation

As the cheetah serves important ecosystem functions as an apex predator, its survival is key to ensuring the healthy functioning of the ecosystems it lives in (Estes et al., 2011). The elimination or disappearance of large carnivores from fragile habitats can have knock‐on effects on other faunal and floral species (Beschta & Ripple, 2009). Threatened by global change and increasing predation pressure from humans, the protection of the last remaining cheetah populations is an imperative part of preserving natural and healthy biodiversity and habitats (Durant et al., 2017). Our findings have several implications for the conservation of cheetahs and highlight the need for further genetic studies and monitoring.

#### 4.3.1 Subspecies‐specific conservation strategies

Even though our genome‐wide nuclear SNP data sample set from East Africa is small (*n* = 3), our results indicate that *A. j. jubatus* and *A. j. raineyi* should be considered distinct at least for the purpose of management and conservation strategies (e.g., in the form of distinct conservation units). Thus, ongoing translocations of southern African cheetahs into parts of East Africa (e.g., Briers‐Louw et al., 2019) should be informed by the conclusion reached in this study and may warrant closer scrutiny by conservationists. Even more so, while the phylogeographic distribution we inferred from our mtDNA data, for the most part, agrees with that described in Charruau et al. (2011), utilizing more samples, we were able to show the presence of two distinct groups within *A. j. raineyi*, with individuals from Tanzania and Kenya showing two different mitochondrial haplogroup assignments, falling either within the haplogroup of *A. j. jubatus* or forming their own haplogroup, here referred to as the *A. j. raineyi* haplogroup. The three individuals from Tanzania (Dobrynin et al., 2015), which form a separate cluster using genome‐wide data (data set 1), show mitochondrial haplotypes that fall within the haplogroup of *A. j. jubatus*. Genome‐wide data for more individuals from East and Northeast Africa will be needed to better resolve the subspecies status in this region, and to better understand the complexities relating nuclear to mitochondrial DNA patterns in cheetahs.

#### 4.3.2 Development of efficient range‐wide genetic monitoring strategies

We generated genome‐wide data for all subspecies, which can function as a baseline for the development of reduced SNP sets, for example, for genotyping using SNP arrays or real‐time PCR, enabling more cost‐effective and large‐scale genetic monitoring. We recently developed a SNP array for *A. j. jubatus* to monitor its legal and illegal trade (Magliolo et al., 2021). However, this approach was only based on individuals from one subspecies. A more generally applicable SNP typing system should include samples from all subspecies. We caution that the presented data set should be extended by more samples of the two *Critically Endangered* subspecies, *A. j. venaticus* and *A. j. hecki*, and of *A. j. raineyi* to avoid ascertainment bias in the selected SNP sets.

#### 4.3.3 The potential for genetic monitoring of illegal trade of cheetahs

Illegal wildlife trade is a major driver of the current biodiversity loss (Maxwell et al., 2016) and affects a large portion of known plant and animal species (Fukushima et al., 2020; Scheffers et al., 2019). Northeast Africa is a poaching hotspot for the illegal cheetah pet trade, mostly to the Gulf states (Nowell, 2014; Tricorache et al., 2018, 2021). It is also probably the region with the greatest negative impact of illegal trade on wild populations of cheetahs (Nowell, 2014). Individuals are probably transported to the Arabian Peninsula via Somalia and Yemen. However, the origins of these animals are poorly known. Information from interdictions and interviews with traders suggest potential origins from opportunistic collections in ethnic Somali regions such as Ethiopia and Kenya (Nowell, 2014; Tricorache et al., 2021). Interestingly, as mentioned above, Northeast Africa is the contact zone between the two subspecies *A. j. soemmeringii* and *A. j. raineyi*. Previous studies along with our current findings indicate the presence of *A. j. soemmeringii* in South Sudan, and the northern and central parts of Ethiopia and Somalia, and of *A. j. raineyi* in Kenya, Tanzania, Uganda, and the southern parts of Somalia and Ethiopia (Charruau et al., 2011; this study). Simple subspecies distinctions for illegally traded individuals and products could thus help us to quantify the respective proportion of the two subspecies in the trade, and ultimately the importance of different Northeast African countries as potential sources of origin. This can then form the basis for targeted programmes to reduce poaching and the illegal wildlife trade of cheetahs in those countries. It will also allow evidence‐based decision‐making for the potential release of confiscated animals into the wild, including identifying appropriate potential founders for subspecies reintroduction attempts into sites where cheetahs are currently extinct, such as Nigeria, Uganda and Rwanda. However, we caution that this aim might be complicated by possible admixture between the two subspecies or incomplete lineage sorting.

#### 4.3.4 Environmental change and immunogenetics

Immunocompetence is an important factor for the survival of a species in changing environments and is influenced by genetic factors such as MHC diversity and the environment in which individuals live (Frankham et al., 2002). For more than two decades, the cheetah has been a popular textbook example for a species with low genetic diversity, especially at MHC loci. Depleted immune gene diversity was previously supported by the cheetah's ability to accept reciprocal skin grafts from unrelated individuals (O'Brien et al., 1983) and by restriction fragment length polymorphism (RLFP) analyses of MHC class I gene transcripts and class II DRB genes (e.g., O'Brien & Yuhki, 1999). However, the findings of low MHC diversity in cheetahs has been debated after Castro‐Prieto, Wachter & Sommer, (2011) and others (e.g., Drake et al., 2004) detected a much higher genetic diversity within MHC I loci compared to previous studies, which they attributed to a larger sample size in their study (149 cheetahs from Namibia; Castro‐Prieto, Wachter & Sommer, 2011). However, they were not able to find any further MHC II‐DRB haplotypes than the four previously described in Drake et al. (2004). Our sampling of 46 individuals, including four of the five classically recognized subspecies, yielded nine MHC II‐DRB haplotypes, with one to four different alleles found within single individuals, similar to Drake et al. (2004) who described two to four alleles per individual. Furthermore, rarefaction analyses indicate the presence of more, yet unsampled, MHC II‐DRB alleles in cheetahs. However, in general, cheetahs show MHC II‐DRB diversities lower than other large felids, such as Bengal tigers (four alleles in 16 individuals; Pokorny et al., 2010), Eurasian lynx (16 alleles in 13 individuals; Wang et al., 2009) and leopards (6 alleles in 25 individuals; Castro‐Prieto et al., 2011). Interestingly, Namibian individuals showed a higher constitutive innate immunity than sympatric leopards, which could be interpreted as a compensation for the potential lack of immunocompetence in the cheetah adaptive immune system due to their lower MHC variability (Heinrich et al., 2017). This is consistent with the lack of substantial evidence of disease events in southern and east African wild cheetah populations, which do not display evidence of compromised immunocompetence (Castro‐Prieto et al., 2011; Schmidt‐Kuntzel et al., 2018).

#### 4.3.5 Reintroductions of cheetahs in Asia

Several reintroduction strategies have been explored over the last years by former cheetah range countries. Frequently, the reasons for cheetah reintroductions include conservation of the species as well as expanded tourism (Boast et al., 2018). Ranjitsinh and Jhala (2010) identified several Indian national parks as potential candidate sites for reintroductions, although all would require extensive preparation and investment before reintroduction could be considered. Genetic studies of regionally extinct populations would be needed to assess past genetic structure and to assign individuals to subspecies. Unfortunately, our sampling only included two individuals from India that were characterized for mtDNA only. While one individual clustered with a sample from Chad, the other one clustered with individuals assigned to *A. j. venaticus* (which is the suspected subspecies for cheetahs from India). Furthermore, the two Indian individuals in Charruau et al. (2011) and Rai et al. (2020) also showed *A. j. venaticus* haplotypes. It is well documented that imports of tamed hunting cheetahs from Northeastern (Pocock, 1939) and Eastern Africa (Divyabhanusinh, 1999) into India and the Arabian Peninsula were a regular occurrence during the European colonial era, which could explain the close relationship of one of our samples to an individual from Chad. Current proposals for cheetah restoration in India (Ranjitsinh & Jhala, 2010) suggest introducing individuals from Africa, as the last remaining *A. j. venaticus* representatives are highly threatened with 50–70 individuals left in the wild (Farhadinia et al., 2017). A small‐scale single‐locus mitochondrial genetic analysis (O'Brien et al., 2017) argued that cheetah subspecies are very closely related and that genetic distances between Asian and African cheetah subspecies are equal to those within Africa. However, our genome‐wide data show that differentiation in cheetahs (average *F*_ST_ of 0.38 for cheetah subspecies) is similar or even higher than that found in other large endangered felids such as tigers (0.11–0.43, Liu et al., 2018), lions (0.16–0.28, Smitz et al., 2018) and leopards (0.05–0.15, Pečnerová et al., 2021), and indicate a distinct genome‐wide differentiation among the African subspecies and also between Asian and African cheetahs, as we found the highest *F*_ST_ values between *A. j. venaticus* and all the African subspecies (ranging from 0.438 to 0.497). Based on our genome‐wide data and in the absence of detailed information on local and regional adaptation in different cheetah subspecies, we advise caution in releasing African cheetahs in Asia. We call for more research on the genetic and ecological differences between subspecies before resorting to introduction of African cheetah subspecies into the historical range of *A. j. venaticus*.

## AUTHOR CONTRIBUTIONS

Stefan Prost and Pamela Anna Burger designed research. Ana Paula Machado, Julia Zumbroich, Lisa Preier, Sarita Mahtani‐Williams, Rene Meissner, Katerina Guschanski, Jaelle C. Brealey, Martin Plasil, Carlos Rodríguez Fernandes and Pamela Anna Burger performed research and analysed data. Carlos Rodríguez Fernandes, Paul Vercammen, Luke T. B. Hunter, Alexei V. Abramov, Petr Horin, Lena Godsall‐Bottriell, Paul Bottriell, Desire Lee Dalton, Antoinette Kotze contributed samples or analytic tools. All authors wrote the manuscript and worked on the revisions.

## CONFLICT OF INTEREST

The authors declare no competing interest.

## BENEFIT‐SHARING STATEMENT

A research collaboration was developed with scientists from the countries providing genetic samples, all collaborators are included as coauthors, the results of research have been shared with the provider communities and the broader scientific community, and the research addresses a priority concern, in this case the conservation of organisms being studied. More broadly, our group is committed to international scientific partnerships, as well as institutional capacity building. All CITES permit numbers are provided in Table S1.

### OPEN RESEARCH BADGES

This article has earned an Open Data Badge for making publicly available the digitally‐shareable data necessary to reproduce the reported results. The data is available at Dryad (https://doi.org/10.5061/dryad.tx95x6b13https://doi.org/10.5061/dryad.tx95x6b13>).

## Supporting information


Figure S1‐S14‐Table S1‐S3‐S4‐S5

Table S2‐S6

## Data Availability

All genomic data are deposited under the BioProject PRJNA624893 on NCBI GenBank. The mitochondrial and MHC alignments can be found on Dryad (https://doi.org/10.5061/dryad.tx95x6b13).
