# Supplementary material for: Genomic analyses show extremely perilous conservation status of African and Asiatic cheetahs (Acinonyx jubatus)
Source: Mol Ecol. 2022 Jul 17;31(16):4208–23. doi: 10.1111/mec.16577 (PMC9540975; doi:10.1111/mec.16577)
Supplement: Supplementary file 1 — Figure S1‐S14‐Table S1‐S3‐S4‐S5 [file MEC-31-4208-s001.docx]

**Supplementary Information for:**

***Genomic Analyses Show Extremely Perilous Conservation Status of African and Asiatic cheetahs (Acinonyx jubatus).***

Stefan Prost, Ana Paula Machado, Julia Zumbroich, Lisa Preier, Sarita Mahtani-Williams, Rene Meissner, Katerina Guschanski, Jaelle C. Brealey, Carlos Rodríguez Fernandes, Paul Vercammen, Luke T. B. Hunter, Alexei V. Abramov, Martin Plasil, Petr Horin, Lena Godsall-Bottriell, Paul Bottriell^†^, Desire Lee Dalton, Antoinette Kotze, Pamela Anna Burger

**Table of Contents:**

| **Materials and Methods** | Page 2 |
| --- | --- |
| **Supplementary Figure S1** | Page 12 |
| **Supplementary Figure S2** | Page 13 |
| **Supplementary Figure S3** | Page 14 |
| **Supplementary Figure S4** | Page 15 |
| **Supplementary Figure S5** | Page 16 |
| **Supplementary Figure S6** | Page 17 |
| **Supplementary Figure S7** | Page 18 |
| **Supplementary Figure S8** | Page 19 |
| **Supplementary Figure S9** | Page 20 |
| **Supplementary Figure S10** | Page 21 |
| **Supplementary Figure S11** | Page 22 |
| **Supplementary Figure S12** | Page 23 |
| **Supplementary Figure S13** | Page 24 |
| **Supplementary Figure S14** | Page 25 |
| ***Supplementary Table S1*** | Page 26 |
| ***Supplementary Table S3*** | Page 27 |
| ***Supplementary Table S4*** | Page 27 |
| ***Supplementary Table S5*** | Page 28 |

**SUPPLEMENTARY METHODS**

**1.1 CITES permits**

Voucher and individual identifiers for samples used in this study can be found in Supplementary Table S2. Samples collected after 1975 were imported under the following CITES numbers: AT 16-E-0753, 16SG006329CR, 15JP001990/TE, 11US761881/9, AT 15-E-1769, D79/DFF. Additionally, we transferred samples between CITES registered institutions (see Supplementary Table S1 for the institution names and their CITES registration code).

**2.1. Datasets descriptions**

**2.1.1. Dataset 1 - nuclear DNA**

Dataset 1 consists of 46 *Acinonyx jubatus* individuals used in the population genomic analyses (belonging to all five classically recognized subspecies) of which we obtained reads from NCBI for three individuals (Genbank: SRR2737543-SRR2737545; from Dobrynin et al. (2015)), 12 *Acinonyx jubatus* individuals of known parent-offspring trios only used for testing the reliability of the relatedness tests, and one specimen of *Puma concolor*. All but the two museum samples were processed using the ddRAD approach following Peterson et al. (2012). The two museum specimens, both of the *Acinonyx j. hecki* subspecies, were sequenced for their whole genomic DNA (as their quality was not high enough to carry out ddRAD processing).

***DNA extraction -*** We extracted DNA from tissue samples of 21 modern cheetahs and one *Puma concolor* specimen using the Quiagen DNeasy Blood and Tissue kit (Qiagen, Venlo, Netherlands) and 32 diluted blood samples using the innuPREP Blood Kit (Analytik Jena AG, Jena, Germany). We extracted the DNA of two museum samples of *A. j. hecki* using the DNA extraction protocol developed by Dabney et al. (2013). The DNA extraction was carried out in a laboratory dedicated to heavily degraded samples at the University of Uppsala in Sweden. This protocol is optimized for retaining short DNA fragments typical for highly degraded historical samples.

***Double-digest RAD sequencing: library preparation and sequencing -*** Double-digest RAD sequencing was carried out for 55 *A. jubatus* samples, and the outgroup species *Puma concolor* by IGA Technologies, Udine, Italy. In brief, *in silico* analysis of the best combination of two restriction enzymes was carried out using Simrad (Lepais and Weir 2014) and the Genbank aciJub1 cheetah genome assembly (GCA_001443585.1; (Dobrynin et al. 2015)). The double-digestion and library preparation was carried out following Peterson et al. (2012), using the SphI and HindIII enzymes. Sequencing was performed on the HiSeq2500 instrument (Illumina, San Diego, CA, USA) using V4 chemistry (2x110bp).

***Genomic DNA: Library preparation and sequencing -*** Illumina sequencing libraries were prepared following the double-indexing strategy of Meyer and Kircher (2010). The samples were pooled in equimolar amounts and sequenced on the Illumina (San Diego, CA, USA) HiSeqX platform (2x150bp) at SciLife, Stockholm, Sweden.

**2.2.1. Dataset 2 - mitochondrial DNA**

We amplified mtDNA regions of 929bp for 56 individuals and 681bp for 57 individuals. We further included 77 individuals from Charruau et al. (2011) in the 681 bp dataset. The mtDNA data was used to infer population structure and included samples of cheetahs from their extant distribution and from regions they have gone extinct from.

***DNA extraction -*** We followed the protocol of Rohland, Siedel, and Hofreiter (2010) for the DNA extraction from museum samples. To avoid DNA contamination, we carried out all extractions in a dedicated laboratory for museum samples at the Research Institute of Wildlife Ecology, Vetmeduni Vienna, Austria.

***DNA amplification and sequencing -*** We targeted two mitochondrial genes that included 14 previously described diagnostic SNPs from Charruau et al. (2011) of the NADH-dehydrogenase subunit 5 (MT-ND5) and the control region (MT-CR). Four sets of primers yielding PCR products between 245 and 375bp were used to amplify a final mtDNA fragment of 929bp for 56 individuals and 681bp for 134 individuals (for primer sequences see Supplementary Table S3). To avoid contamination, PCR reactions were prepared in a separate laboratory and the DNA extractions were added to the solutions in another room. Negative controls were included in each PCR reaction. The purified PCR products were sent for Sanger sequencing in both directions at Macrogen Europe Inc. (Amsterdam, Netherlands) and LCG Genomics (Berlin, Germany).

**2.3.1. Dataset 3 - Mini-barcodes**

We designed a mini-barcode approach to investigate whether all *A. j. soemmeringii* carry the 3 bp deletion in the MT-ND5 gene described in Charruau et al. (2011), and as a quick subspecies assignment test.

***DNA extraction -*** Here we used the extracts of all 15 *A. j. soemmeringii* samples described in Dataset 1.

***DNA amplification and sequencing -*** We designed three overlapping mini-amplicons that amplify a total of 190bp, based on diagnostic sites inferred from our mitochondrial haplotype data for ND5 and control region. Primer sequences can be found in Supplementary Table S3. We then sequenced these mini-barcodes from 12 *A. j. soemmeringii* individuals for which we obtained ddRAD data on an Illumina iSeq 100 (2x150bp) following the two step sequencing protocol of Lange et al. (2014).

**2.4.1. Dataset 4 - MHC**

Here we sequenced the MHC class II DRB exon 2 of 46 individuals (belonging to four of the five classical subspecies; all but *A. j. hecki*) to investigate the immunogenetics of cheetahs.

***DNA extraction -*** We used the Qiagen DNeasy Blood and Tissue kit for DNA extraction from hair and tissue samples, and the VWR PeqGold™ Tissue DNA Mini Kit Plus for blood samples.

***DNA amplification and sequencing -*** We carried out PCR amplification of the target region as described in Castro-Prieto, Wachter, and Sommer (2011) using the primers: DRB_SL-F (GCGTCAGTGTCTTCCAGGAG) and DRB_SL-R (GGGACCCAGTCTCTGTCTCA). Indexing, multiplexing and sequencing was carried out following the Illumina Nextera XT DNA LibraryPrep reference guide. Sequencing was performed on an Illumina MiSeq (2x250bp).

**3. Analyses**

**3.1.1. Raw data processing (dataset 1)**

First, we assessed the raw read quality using FastQC (https://www.bioinformatics.babraham.ac.uk/projects/fastqc/). Read pairs per sample ranged from 120,739 to 38.9 million, with an average of 3.58 million for the ddRAD samples. Genome sequencing of the two *A. j. hecki* samples resulted in 175.96 million and 127.59 million read pairs. GC content for the ddRAD and the genomic reads ranged from 41% to 43%. The average read Phred score was 36 for the ddRAD samples and 40 for the genomic reads. Next, we mapped the read data against the Aci_jub_2 cheetah genome assembly (GCA_003709585.1) using BWA mem (version 0.7.17-r1188) (Li and Durbin 2010) and processed the resulting mapping files using samtools (version 1.9) (Li et al. 2009). We subsequently assessed the mapping quality using Qualimap (Okonechnikov, Conesa, and García-Alcalde 2016). The coverage ranged from 0.9x to 217x for the ddRAD data. For the two museum samples we further carried out adapter-trimming and duplicate removal using AdapterRemoval2 (Schubert, Lindgreen, and Orlando 2016) and Picard (<https://broadinstitute.github.io/picard/>) after the mapping and processing using BWA mem(version 0.7.17-r1188) and samtools (version 1.9). The two *A. j. hecki* samples showed average coverages of 3.8x and 4.9x, respectively.

**3.1.2. SNP filtering (dataset 1)**

The resulting mapping files were then processed using ANGSD (Korneliussen, Albrechtsen, and Nielsen 2014), which was specifically developed for population genomic analyses of low coverage data. First, we carried out filtering using SNPcleaner (v 2.24) (Fumagalli et al. 2014). To do so, we created a vcf file for all samples using samtools (version 1.9). We then filtered the vcf file for (a) the presence of no more than 25% of missing data for individual sites, (b) a maximum coverage of 120x per individual to avoid calling sites in highly repetitive regions, and (c) a minimum coverage of 3x for each individual. Next, we extracted the first two columns of the resulting bed file to generate the filtered SNP files for the subsequent ANGSD analyses. This resulted in 3,743 SNPs.

We included 46 individuals in the population genomic analyses (43 from this study and three individuals from Dobrynin et al. (2015)). Genotype likelihoods and genotype calls for the downstream analyses were inferred using ANGSD. We required sites to have a minimum base quality score of 20 (-minQ 20) and also considered reads where only one of the pair mapped (-only_proper_pairs 0; recommended for low coverage data). We specified the 3,743 SNPs in ANGSD using the *-sites* option, and provided the genome reference using *-anc* and *-ref*.

**3.2.1. Population differentiation (dataset 1)**

The following analyses were carried out with the 46 individuals from dataset 1 (excluding the 12 individuals only used for the relatedness testing assessment). First, we carried out principal component analyses using ANGSD and pcangsd (Meisner and Albrechtsen 2018). For this analysis we generated the required genotype likelihood file in beagle format using options: *-GL 1 -doGlf 2 -doMajorMinor 1 -doMaf 2 -SNP_pval 1e-6*. This file was then analysed using pcangsd. Next, we investigated population differentiation using *F*_ST_. As some estimators of *F*_ST_ can be biased by uneven sampling (see e.g., Willing et al. 2012), we created three replicates for which we randomly subsampled *A. j. jubatus* and *A. j.* *soemmeringii* down to three individuals. We calculates *F*_ST_ values using ANGSD (options: *-GL 1 -doSaf 1*) and *realSFS* (*realSFS fst index* and *realSFS fst stats*). To further investigate the reliability of our *F*_ST_ estimates, we carried out a randomization test. To do so, we generated three data sets, mimicking our original sampling (four groups of three individuals and one group of two individuals), in which each group was made up of randomly selected individuals of different subspecies. We then checked the *F*_ST_ distributions, the random ones and the one obtained from the original set for normality with the Shapiro-Wilk test in the R (3.4.3.) *stats* package. As both followed a normal distribution, we next tested whether both distributions had the same variance using the *var.test()* function of the *stats* package. This showed that both distributions are independent, and we thus proceeded with an F-Test (implemented in the *stats* package) to see whether both were significantly different from each other. Next, we looked for signatures of admixture using ANGSD (using options: *-GL 1 -doGlf 2 -doMajorMinor 1 -SNP_pval 1e-6 -doMaf 1*) and ngsAdmix (Skotte, Korneliussen, and Albrechtsen 2013). Again, in order to avoid sampling biases, due to the different numbers of samples per subspecies, we generated different sets of three randomly chosen individuals for each subspecies (except for *A. j. raineyi*, *A. j. venaticus* and *A. j. hecki* where we only had three, three and two individuals in our sampling, respectively). We further carried out a separate ngsAdmix analysis restricted to all individuals of the two subspecies *A. j. soemmeringii* and *A. j. jubatus*. We carried out 50 replicates for all ngsAdmix runs ranging from k=2 to k=5. The results were analysed and visualized using CLUPMACK (Kopelman et al. 2015). Lastly, we performed an EEMS (Petkova et al. 2016) analysis to infer effective migration rates for the African cheetah subspecies. To do so, we first ran ANGSD (using options: *-GL 1 -doMaf 1 -doMajorMinor 1 -doIBS 1 -doCounts 1 -makeMatrix 1*). Then we ran EEMS with 400 demes and an MCMC chain of 5,000,000, with a burn in of 1,000,000 and selecting each 9,999 iterations. We defined the coordinates of the geographic area manually using the tool: http://www.birdtheme.org/useful/v3tool.html. As we do not have exact geographic locations for the samples, we selected the center of the current distribution of the subspecies within the respective country. To plot the results, we used the make_eems_plots script (available here: https://github.com/dipetkov/reemsplots2) as described in Pečnerová et al. 2021.

**3.2.2. Phylogenetic analyses (dataset 1)**

For dataset 1, we carried out phylogenetic analyses using three different methods: (1) using genetic distances, (2) a phylogenetic network approach and (3) a maximum likelihood based analysis using genotype calls. (1) For the genetic distance approach we applied a combination of ANGSD (options: *-GL 1 -doMaf 2 -doMajorMinor 1 -doGeno 8 -doPost 1 -doSaf 1 -fold 1 -SNP_pval 1e-6*), ngsDist (Fumagalli et al. 2014) (options: *--n_boot_rep 100 --boot_block_size 1*) and FastME (Lefort, Desper, and Gascuel 2015). Bootstrap support values were placed on the tree using RaxML (Stamatakis 2014). (2) For the phylogenetic network inference, we first used ANGSD (*-GL 1 -doMaf 2 -doMajorMinor 1 -doGeno 2 -doPost 1 -doSaf 1 -fold 1 -SNP_pval 1e-6*) to generate genotypes and genotype probabilities. Next, we used PopGenTools (<https://github.com/CGRL-QB3-UCBerkeley/PopGenTools>) to convert the genotypes to the adegenet input format to calculate genetic distances using the R package adegenet (Jombart and Ahmed 2011). We visualized the phylogenetic network topology using Splitstree (Huson, Kloepper, and Bryant 2008). (3) For the maximum likelihood-based analysis we generated consensus sequences from the mapping files using ANGSD (-*doFasta 3*). Then, we extracted the regions around the 3,743 SNPs (about 100bp on each side). Next, we concatenated the 200bp fragments, aligned them using MAFFT (Katoh & Standley, 2013) and removed sites that showed >60% of missing data. We then calculated the ML phylogeny using IQ-Tree (Nguyen et al. 2015), using the option -m TEST to have IQ-Tree selected the best fitting substitution model. We performed 10,000 bootstrap replicates and 10,000 SH-like approximate likelihood ratio tests.

**3.2.3. Conservation genomic parameters (dataset 1)**

Here we investigated classical conservation genomic parameters, such as inbreeding, relatedness and heterozygosity. Inbreeding was inferred using two approaches: (1) ANGSD (*-GL 1 -doMaf 2 -doMajorMinor 1 -doGeno 32 -doPost 1 -doSaf 1 -doGlf 3 -doCounts 1 -SNP_pval 1e-6*), ngsF (Fumagalli et al. 2014) (step 1: *--min_epsilon 1e-9 --approx_EM --seed 0 -init_value r --max_iters 500*, and step2: *--min_epsilon 1e-9 --seed 0 --max_iters 1500*, using initial values from step 1 provided with the option: *-init_value*), and (2) ANGSD (options: *-GL 1 -doGlf 3 -doMajorMinor 1 -SNP_pval 1e-6 -doMaf 1*) and ngsRelate (v2) (Hanghøj et al. 2019). We further used ngsRelate (v1 (Korneliussen and Moltke 2015) and v2 (Hanghøj et al. 2019) which corrects for inbreeding) to carry out relatedness analyses. In order to check their performance, we first ran the relatedness analysis tools using only the 12 individuals consisting of four parent-offspring trios (IDs: 448-459). Next, we carried out heterozygosity analyses using ANGSD (*-GL 1 -doCounts 1 -dosaf 1 -fold 1 -minmapq 30 -C 50*; we set the minimum coverage to 3x using *-minIndDepth*) and realSFS (part of the ANGSD package). Here, we did not restrict our analyses to filtered sites, to be able to compare our estimates to published genome-wide heterozygosity values of other felids. Due to low coverage and quality, typical for degraded museum DNA, we removed the two museum samples from this analysis.

**3.2.4. Modern and historical cheetah population structure using mitochondrial DNA (dataset 2)**

For the mitochondrial DNA data we aligned all sequences using Codon Code Aligner v3.0.2 (Codon Code Corporation). We obtained the reference sequence for the cheetah mitochondrial genome from GenBank (accession number NC_005212.1 (Burger et al. 2004)). We carried out parallel analyses on the two concatenated datasets that differed in the length of the fragment and the number of individuals (Supplementary Table S2). The first analysis comprised the largest mtDNA fragment of 929 bp amplified in 58 individuals. The second line of analysis incorporated the alignment of 78 individuals from Charruau et al. (2011) and 57 generated in this study (681bp). Median-joining networks were created using the freely available software tool Popart (Leigh and Bryant 2015). We investigated the molecular variance in our data using AMVOA analysis, implemented in the software Arleguin 3.5 (Excoffier and Lischer 2010). Furthermore, we investigated the homology of the mtDNA fragments to known NUMTs in the published cheetah genome (GCA_003709585.1) using the default settings in blastn (the “somewhat similar sequence” modus).

**3.2.5. Testing for the presence of the 3 bp deletion in the MT-ND5 gene in *A. j. soemmeringii* (dataset 3)**

We aligned all sequences using Mafft (Katoh & Standley, 2013). Next, we generated median joining networks for the mini-barcode data using Popart (Leigh and Bryant 2015).

**3.2.6. Adaptive immune system diversity in cheetahs (dataset 4)**

We mapped the reads against the MHC class II DRB exon 2 reference sequence from Castro-Pietro et al. (2011) using BWA-MEM, and further processed the mapping file using Samtools. We called variants using Picard v. 2.8.2 (http://broadinstitute.github.io/picard) and GATK (v.3.1.8) (McKenna et al. 2010). The main alleles were phased using the FastaAlternateReferenceMaker command in GATK, based on a minimum of 6 reads per sample, and haplotypes were verified manually by visualization of the coordinate-sorted BAM files using IGViewer (Robinson et al. 2017). We estimated haplotype diversity (Hd) and nucleotide diversity (π) using DNAsp (Librado and Rozas 2009). Rarefaction analyses were carried out using EstimateS (Colwell and Elsensohn, 2014).

**4. References**

Burger, Pamela A, Ralf Steinborn, Christian Walzer, Thierry Petit, Mathias Mueller, and Franz Schwarzenberger. 2004. “Analysis of the Mitochondrial Genome of Cheetahs (Acinonyx Jubatus) with Neurodegenerative Disease.” *Gene* 338 (1): 111–19. https://doi.org/10.1016/j.gene.2004.05.020.

Castro-Prieto, Aines, Bettina Wachter, and Simone Sommer. 2011. “Cheetah Paradigm Revisited: MHC Diversity in the World’s Largest Free-Ranging Population.” *Molecular Biology and Evolution* 28 (4): 1455–68. https://doi.org/10.1093/molbev/msq330.

Charruau, P., C. Fernandes, P. OROZCO‐terWENGEL, J. Peters, L. Hunter, H. Ziaie, A. Jourabchian, et al. 2011. “Phylogeography, Genetic Structure and Population Divergence Time of Cheetahs in Africa and Asia: Evidence for Long-Term Geographic Isolates.” *Molecular Ecology* 20 (4): 706–24. https://doi.org/10.1111/j.1365-294X.2010.04986.x.

Colwell, R.K., & Elsensohn, J. E. (2014). EstimateS turns 20: statistical estimation of species richness and shared species from samples, with non‐parametric extrapolation. *Ecography*, *37*(6), 609-613.

Dabney, Jesse, Michael Knapp, Isabelle Glocke, Marie-Theres Gansauge, Antje Weihmann, Birgit Nickel, Cristina Valdiosera, et al. 2013. “Complete Mitochondrial Genome Sequence of a Middle Pleistocene Cave Bear Reconstructed from Ultrashort DNA Fragments.” *Proceedings of the National Academy of Sciences* 110 (39): 15758–63. https://doi.org/10.1073/pnas.1314445110.

Dobrynin, Pavel, Shiping Liu, Gaik Tamazian, Zijun Xiong, Andrey A. Yurchenko, Ksenia Krasheninnikova, Sergey Kliver, et al. 2015. “Genomic Legacy of the African Cheetah, Acinonyx Jubatus.” *Genome Biology* 16 (1): 277. https://doi.org/10.1186/s13059-015-0837-4.

Excoffier, L., & Lischer, H. E. (2010). Arlequin suite ver 3.5: a new series of programs to perform population genetics analyses under Linux and Windows. Molecular ecology resources, 10(3), 564-567.

Fumagalli, Matteo, Filipe G. Vieira, Tyler Linderoth, and Rasmus Nielsen. 2014. “NgsTools: Methods for Population Genetics Analyses from next-Generation Sequencing Data.” *Bioinformatics* 30 (10): 1486–87. https://doi.org/10.1093/bioinformatics/btu041.

Hanghøj, Kristian, Ida Moltke, Philip Alstrup Andersen, Andrea Manica, and Thorfinn Sand Korneliussen. 2019. “Fast and Accurate Relatedness Estimation from High-Throughput Sequencing Data in the Presence of Inbreeding.” *GigaScience* 8 (5). https://doi.org/10.1093/gigascience/giz034.

Huson, Daniel H, Tobias Kloepper, and David Bryant. 2008. “SplitsTree 4.0 - Computation of Phylogenetic Trees and Networks.” *2008* 14: 68–73.

Jombart, Thibaut, and Ismaïl Ahmed. 2011. “Adegenet 1.3-1: New Tools for the Analysis of Genome-Wide SNP Data.” *Bioinformatics* 27 (21): 3070–71. https://doi.org/10.1093/bioinformatics/btr521.

Katoh, K., & Standley, D. M. (2013). MAFFT Multiple SequenceAlignment Software Version 7: Improvements in performance and usability. Molecular Biology and Evolution, 30, 772–780. https://doi.org/10.1093/molbe v/mst010

Kopelman, Naama M., Jonathan Mayzel, Mattias Jakobsson, Noah A. Rosenberg, and Itay Mayrose. 2015. “Clumpak: A Program for Identifying Clustering Modes and Packaging Population Structure Inferences across K.” *Molecular Ecology Resources* 15 (5): 1179–91. https://doi.org/10.1111/1755-0998.12387.

Korneliussen, Thorfinn Sand, Anders Albrechtsen, and Rasmus Nielsen. 2014. “ANGSD: Analysis of Next Generation Sequencing Data.” *BMC Bioinformatics* 15 (1): 356. https://doi.org/10.1186/s12859-014-0356-4.

Korneliussen, Thorfinn Sand, and Ida Moltke. 2015. “NgsRelate: A Software Tool for Estimating Pairwise Relatedness from next-Generation Sequencing Data.” *Bioinformatics* 31 (24): 4009–11. https://doi.org/10.1093/bioinformatics/btv509.

Lange, Vinzenz, Irina Böhme, Jan Hofmann, Kathrin Lang, Jürgen Sauter, Bianca Schöne, Patrick Paul, et al. 2014. “Cost-Efficient High-Throughput HLA Typing by MiSeq Amplicon Sequencing.” *BMC Genomics* 15 (1): 63. https://doi.org/10.1186/1471-2164-15-63.

Lefort, Vincent, Richard Desper, and Olivier Gascuel. 2015. “FastME 2.0: A Comprehensive, Accurate, and Fast Distance-Based Phylogeny Inference Program.” *Molecular Biology and Evolution* 32 (10): 2798–2800. https://doi.org/10.1093/molbev/msv150.

Leigh, Jessica W., and David Bryant. 2015. “Popart: Full-Feature Software for Haplotype Network Construction.” *Methods in Ecology and Evolution* 6 (9): 1110–16. https://doi.org/10.1111/2041-210X.12410.

Lepais, Olivier, and Jason T. Weir. 2014. “SimRAD: An R Package for Simulation-Based Prediction of the Number of Loci Expected in RADseq and Similar Genotyping by Sequencing Approaches.” *Molecular Ecology Resources* 14 (6): 1314–21. https://doi.org/10.1111/1755-0998.12273.

Li, Heng, and Richard Durbin. 2010. “Fast and Accurate Long-Read Alignment with Burrows–Wheeler Transform.” *Bioinformatics* 26 (5): 589–95. https://doi.org/10.1093/bioinformatics/btp698.

Li, Heng, Bob Handsaker, Alec Wysoker, Tim Fennell, Jue Ruan, Nils Homer, Gabor Marth, Goncalo Abecasis, and Richard Durbin. 2009. “The Sequence Alignment/Map Format and SAMtools.” *Bioinformatics* 25 (16): 2078–79. https://doi.org/10.1093/bioinformatics/btp352.

Librado, P., and J. Rozas. 2009. “DnaSP v5: A Software for Comprehensive Analysis of DNA Polymorphism Data.” *Bioinformatics* 25 (11): 1451–52. https://doi.org/10.1093/bioinformatics/btp187.

McKenna, Aaron, Matthew Hanna, Eric Banks, Andrey Sivachenko, Kristian Cibulskis, Andrew Kernytsky, Kiran Garimella, et al. 2010. “The Genome Analysis Toolkit: A MapReduce Framework for Analyzing next-Generation DNA Sequencing Data.” *Genome Research* 20 (9): 1297–1303. https://doi.org/10.1101/gr.107524.110.

Meisner, Jonas, and Anders Albrechtsen. 2018. “Inferring Population Structure and Admixture Proportions in Low-Depth NGS Data.” *Genetics* 210 (2): 719–31. https://doi.org/10.1534/genetics.118.301336.

Meyer, Matthias, and Martin Kircher. 2010. “Illumina Sequencing Library Preparation for Highly Multiplexed Target Capture and Sequencing.” *Cold Spring Harbor Protocols* 2010 (6): pdb.prot5448. https://doi.org/10.1101/pdb.prot5448.

Nguyen, Lam-Tung, Heiko A. Schmidt, Arndt von Haeseler, and Bui Quang Minh. 2015. “IQ-TREE: A Fast and Effective Stochastic Algorithm for Estimating Maximum-Likelihood Phylogenies.” *Molecular Biology and Evolution* 32 (1): 268–74. https://doi.org/10.1093/molbev/msu300.

Okonechnikov, Konstantin, Ana Conesa, and Fernando García-Alcalde. 2016. “Qualimap 2: Advanced Multi-Sample Quality Control for High-Throughput Sequencing Data.” *Bioinformatics* 32 (2): 292–94. <https://doi.org/10.1093/bioinformatics/btv566>.

Pečnerová, P., Garcia-Erill, G., Liu, X., Nursyifa, C., Waples, R. K., Santander, C. G., Quinn, L., Frandsen, P., Meisner, J., Stæger, F. F., Rasmussen, M. S., Brüniche-Olsen, A., Hviid Friis Jørgensen, C., da Fonseca, R. R., Siegismund, H. R., Albrechtsen, A., Heller, R., Moltke, I., & Hanghøj, K. (2021). High genetic diversity and low differentiation reflect the ecological versatility of the African leopard. *Current Biology*. https://doi.org/10.1016/j.cub.2021.01.064

Peterson, Brant K., Jesse N. Weber, Emily H. Kay, Heidi S. Fisher, and Hopi E. Hoekstra. 2012. “Double Digest RADseq: An Inexpensive Method for De Novo SNP Discovery and Genotyping in Model and Non-Model Species.” *PLoS ONE* 7 (5). https://doi.org/10.1371/journal.pone.0037135.

Petkova, D., Novembre, J., & Stephens, M. (2016). Visualizing spatial population structure with estimated effective migration surfaces. *Nature genetics*, *48*(1), 94-100.

Robinson, James T., Helga Thorvaldsdóttir, Aaron M. Wenger, Ahmet Zehir, and Jill P. Mesirov. 2017. “Variant Review with the Integrative Genomics Viewer.” *Cancer Research* 77 (21): e31–34. https://doi.org/10.1158/0008-5472.CAN-17-0337.

Rohland, Nadin, Heike Siedel, and Michael Hofreiter. 2010. “A Rapid Column-Based Ancient DNA Extraction Method for Increased Sample Throughput.” *Molecular Ecology Resources* 10 (4): 677–83. https://doi.org/10.1111/j.1755-0998.2009.02824.x.

Schubert, Mikkel, Stinus Lindgreen, and Ludovic Orlando. 2016. “AdapterRemoval v2: Rapid Adapter Trimming, Identification, and Read Merging.” *BMC Research Notes* 9 (1): 88. https://doi.org/10.1186/s13104-016-1900-2.

Skotte, Line, Thorfinn Sand Korneliussen, and Anders Albrechtsen. 2013. “Estimating Individual Admixture Proportions from Next Generation Sequencing Data.” *Genetics* 195 (3): 693–702. https://doi.org/10.1534/genetics.113.154138.

Stamatakis, Alexandros. 2014. “RAxML Version 8: A Tool for Phylogenetic Analysis and Post-Analysis of Large Phylogenies.” *Bioinformatics* 30 (9): 1312–13. https://doi.org/10.1093/bioinformatics/btu033.

**Supplementary Figures**

**Supplementary Figure S1: PCA analyses for the genome-wide SNP data (PC1 versus PC3).** Blue: *A. j. venaticus*, green: *A. j. hecki*, grey: *A. j. soemmeringii,* purple: *A. j. jubatus,* or
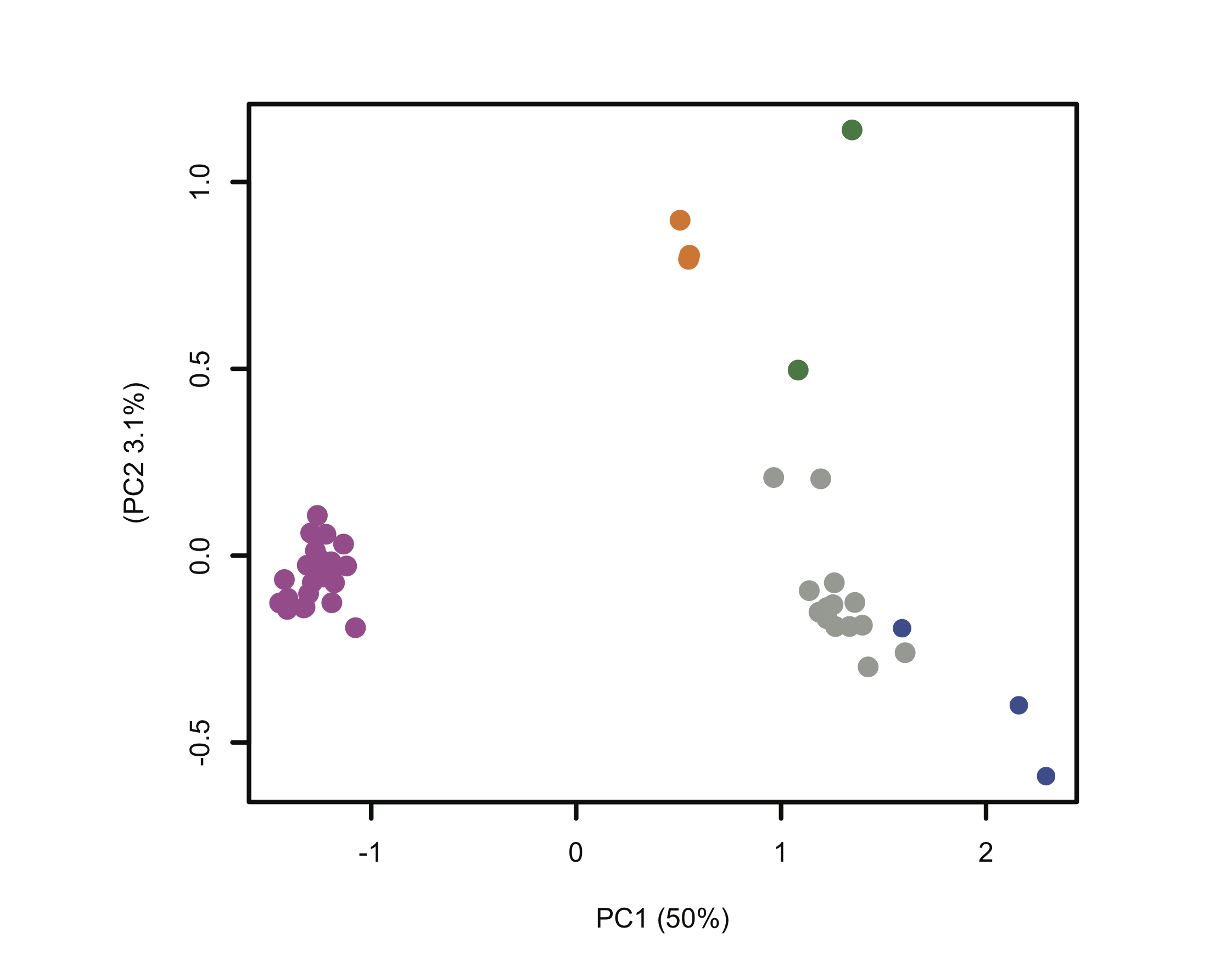
ange: *A. j. raineyi*.

**Supplementary Figure S2: Maximum likelihood based phylogenetic tree reconstruction based on genotype calls for the 3,743 SNPs. B**ootstrapping and the SH-like approximate likelihood ratio test were carried out for 1,000 replicates. Blue: *A. j. venaticus*, green: *A. j. hecki*, grey: *A. j. soemmeringii,* purple: *A. j. jubatus,* orange: *A. j. raineyi*. We used the Puma (*Puma concolor*) as outgroup.


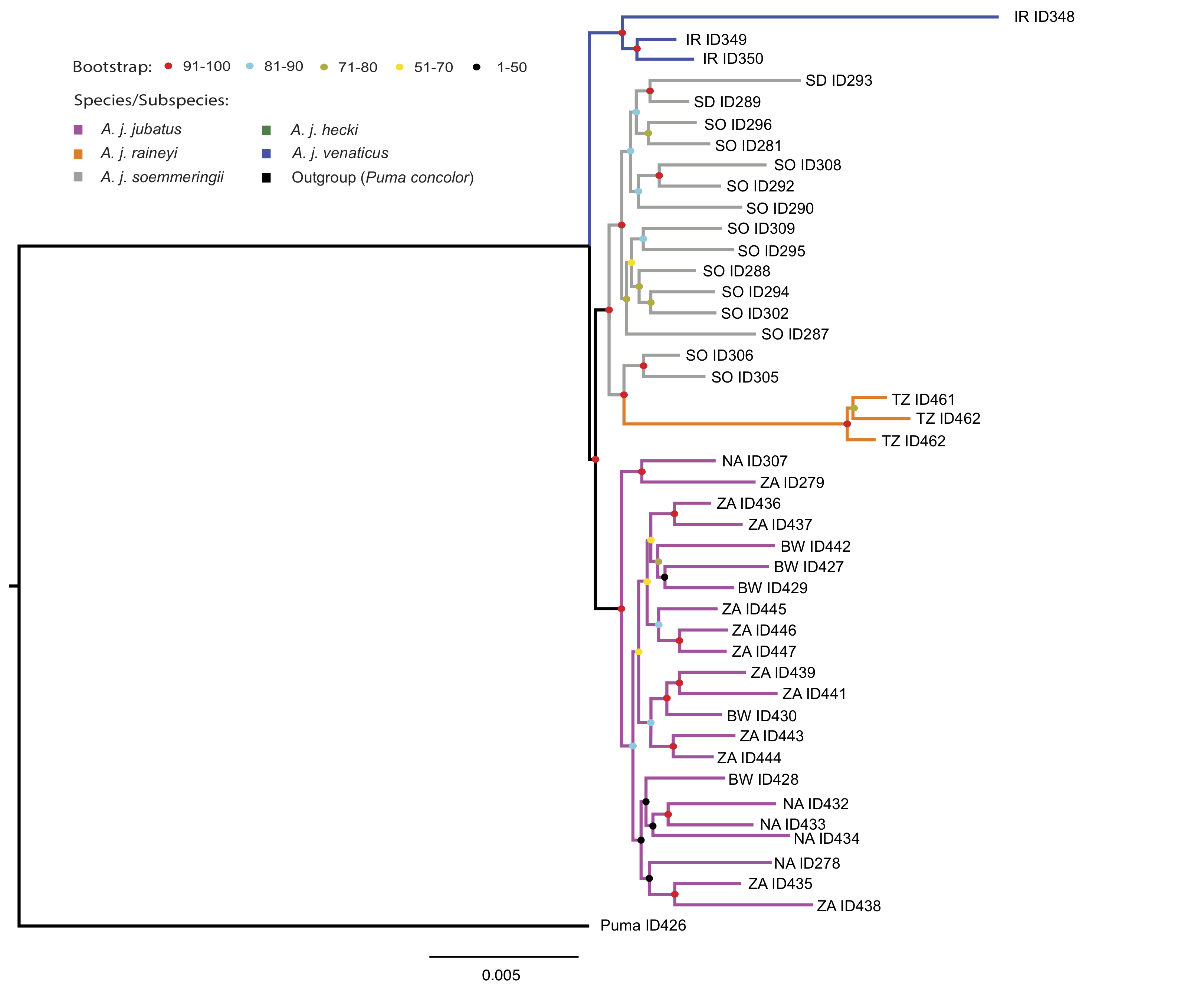


**Supplementary Figure S3: Phylogenetic relationships of representatives for the five clas**
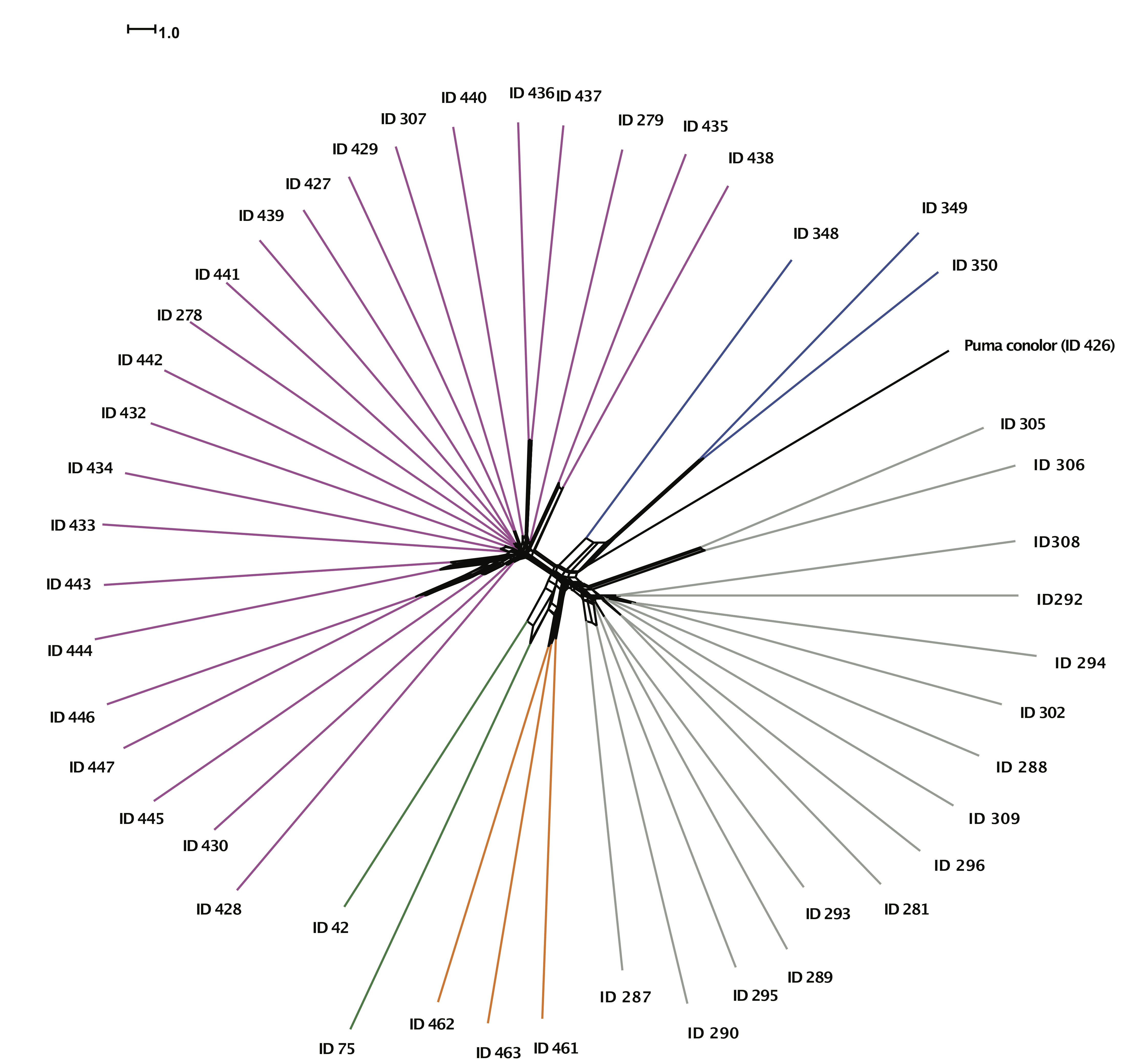
**sical cheetah subspecies using the phylogenetic network approach implemented in SplitsTree.** Blue: *A. j. venaticus*, green: *A. j. hecki*, grey: *A. j. soemmeringii,* purple: *A. j. jubatus,* orange: *A. j. raineyi*. We used the Puma (*Puma concolo*r) as outgroup.

**Supplementary Figure S4: Admixture results for the three independent replicate runs.** Results are shown for 50 replicates of K=2, K=3, K=4 and K=5. Each run used different individuals for *A. j. jubatus* and *A. j. soemmeringii*. Only groupings supported by more than four replicates are shown.


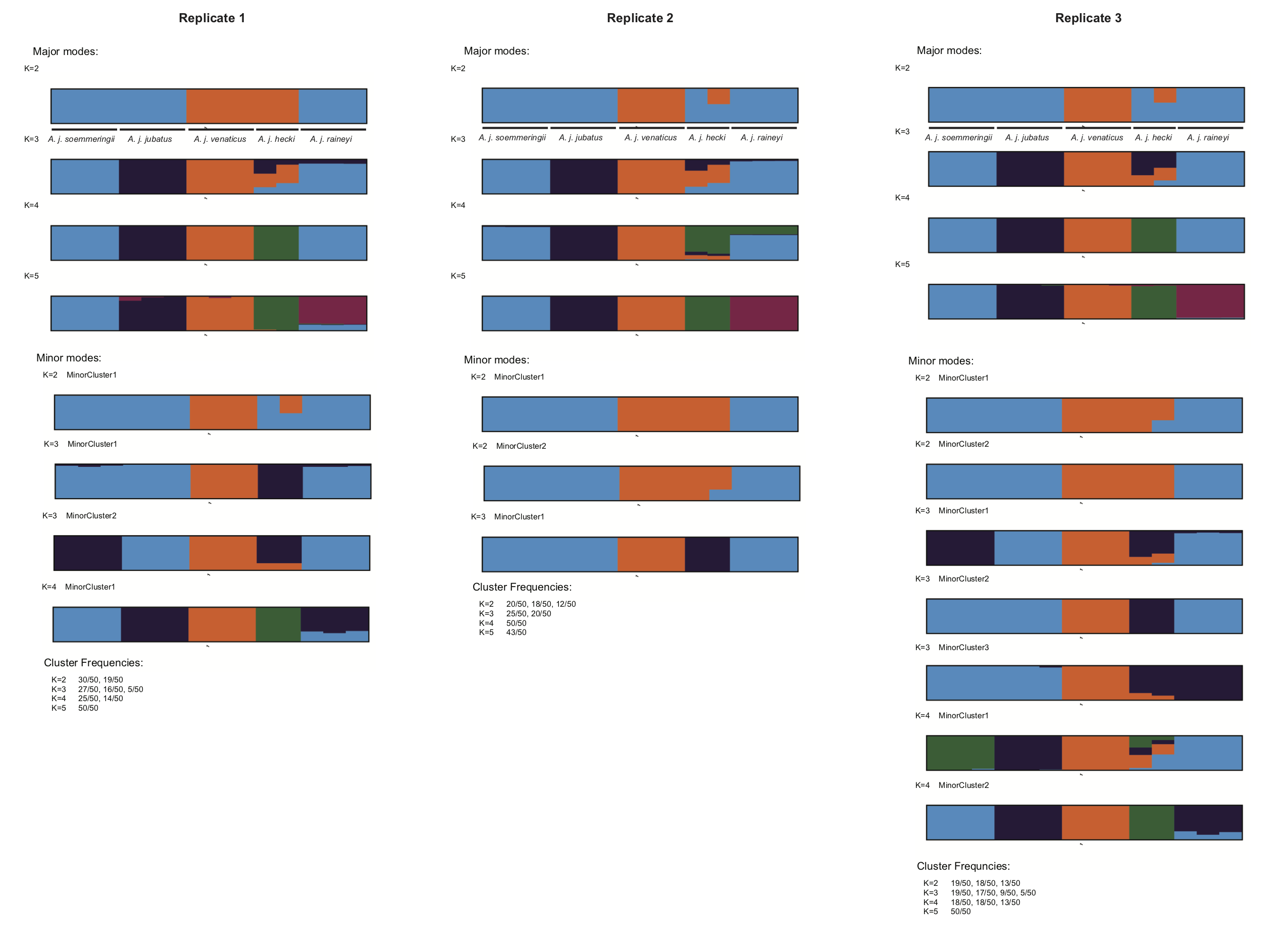


**Supplementary Figure S5: Evaluation of the model fit based on the inferred admixture** **proportions.** Results are shown for K=2, K=3, K=4 and K=5.


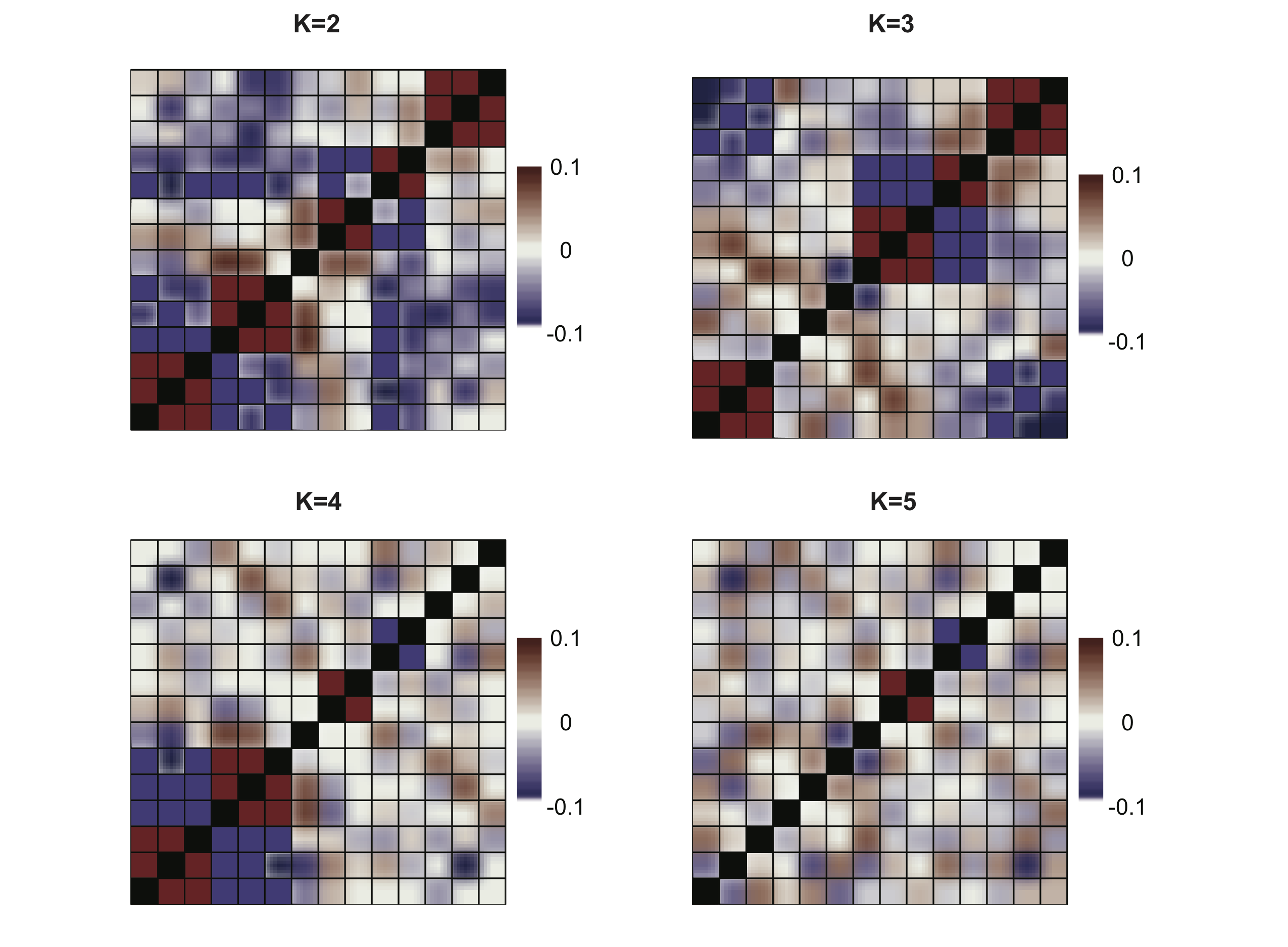


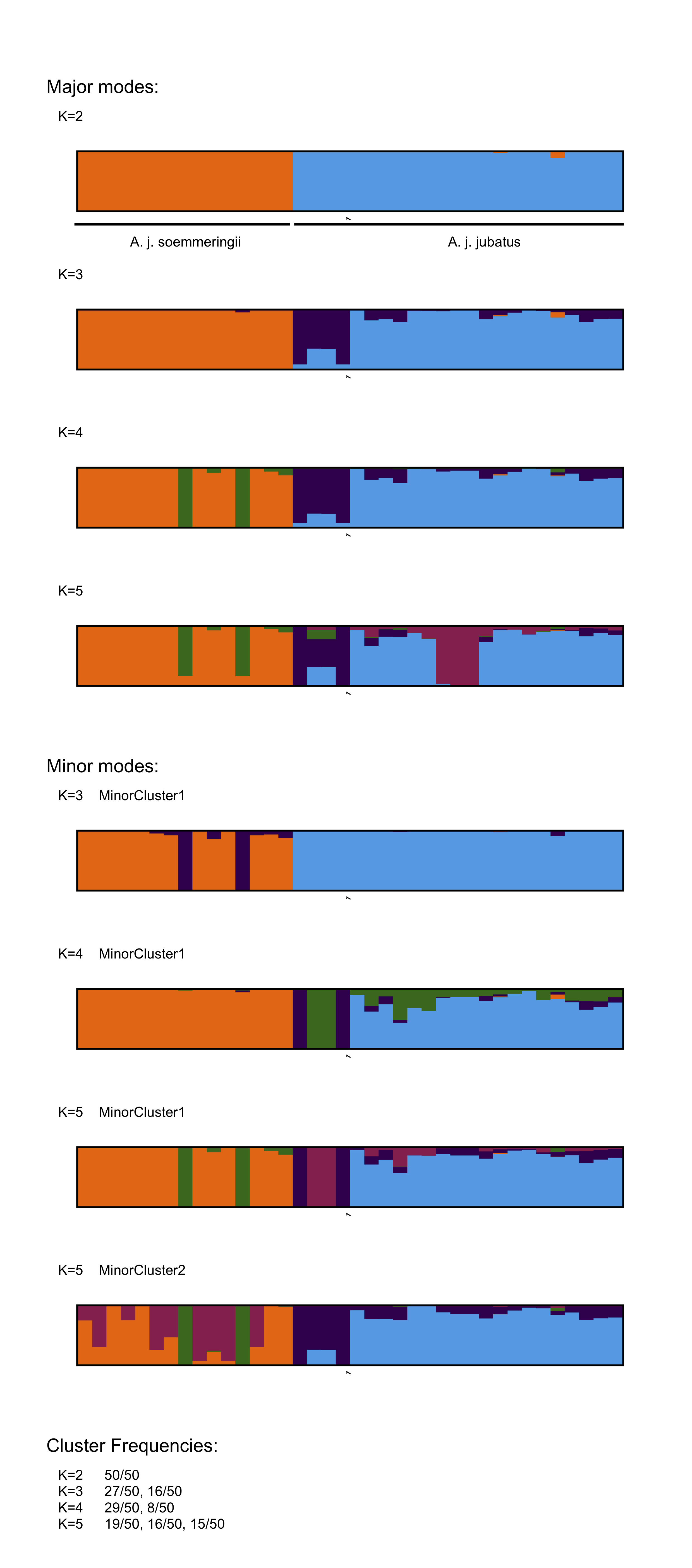
**Supplementary Figure S6: Admixture results for the *A. j. jubatus* and *A. j. soemmeringii* dataset.** Results are shown for 50 replicates of K=2, K=3, K=4 and K=5. Only groupings supported by more than four replicates are shown.

**Supplementary Figure S7: Inbreeding values calculated using the method of [21].** Blue: *A. j. venaticus*, green: *A. j. hecki*, grey: *A. j. soemmeringii,* purple: *A. j. jubatus,* orange: *A. j. raineyi*.


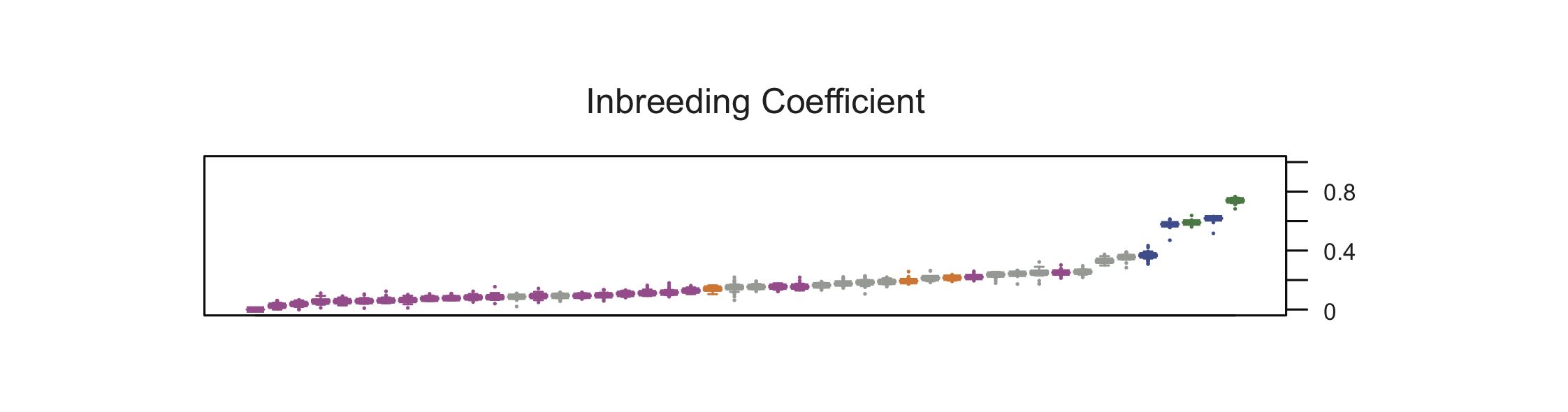


**Supplementary Figure S8: Plot showing K0 versus the coefficient of relatedness (r) for *A. j. jubatus* and *A. j. soemmeringii.*** The expected relatedness are shown for different areas in the plot. PO…parent offspring, Sib…sibling, 2nd…second generation cousins, 3rd…third generation cousins, 4th…fourth generation cousins and UR…unrelated. Grey: *A. j. soemmeringii* and purple: *A. j. jubatus.*


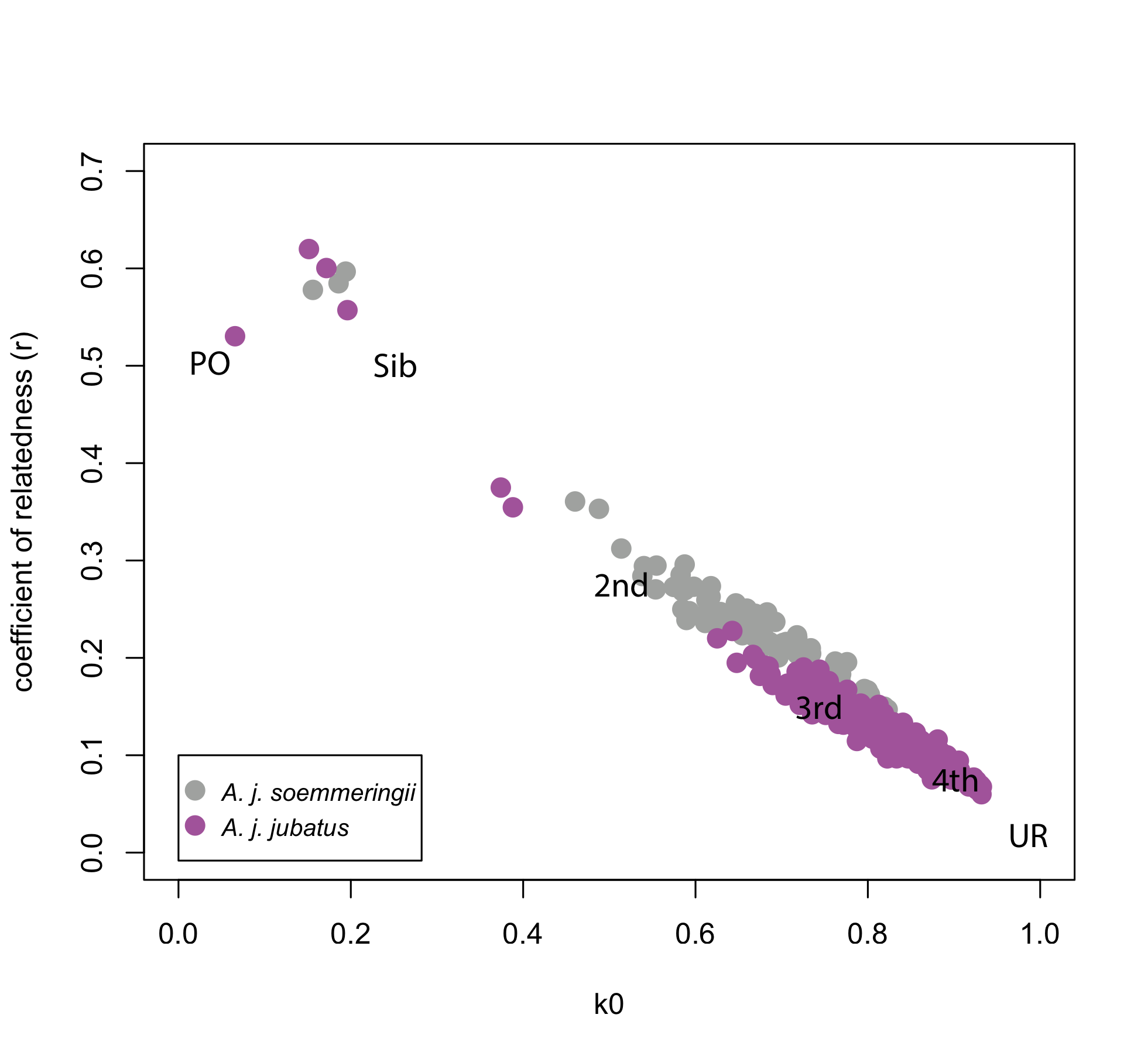


**Supplementary Figure S9: Relatedness (K2) of four parent-offspring trios.** Top panel: relatedness inferred without accounting for inbreeding, and bottom panel relatedness inferred after correcting for inbreeding. Darker blue indicates a closer relationship and white color indicates no relationship between individuals. The four trios are made up of (1) individuals: 1,2 and 3, (2) individuals: 4,5,6, (3) individuals: 7, 8 and 9, and (4) individuals: 10, 11 and 12.


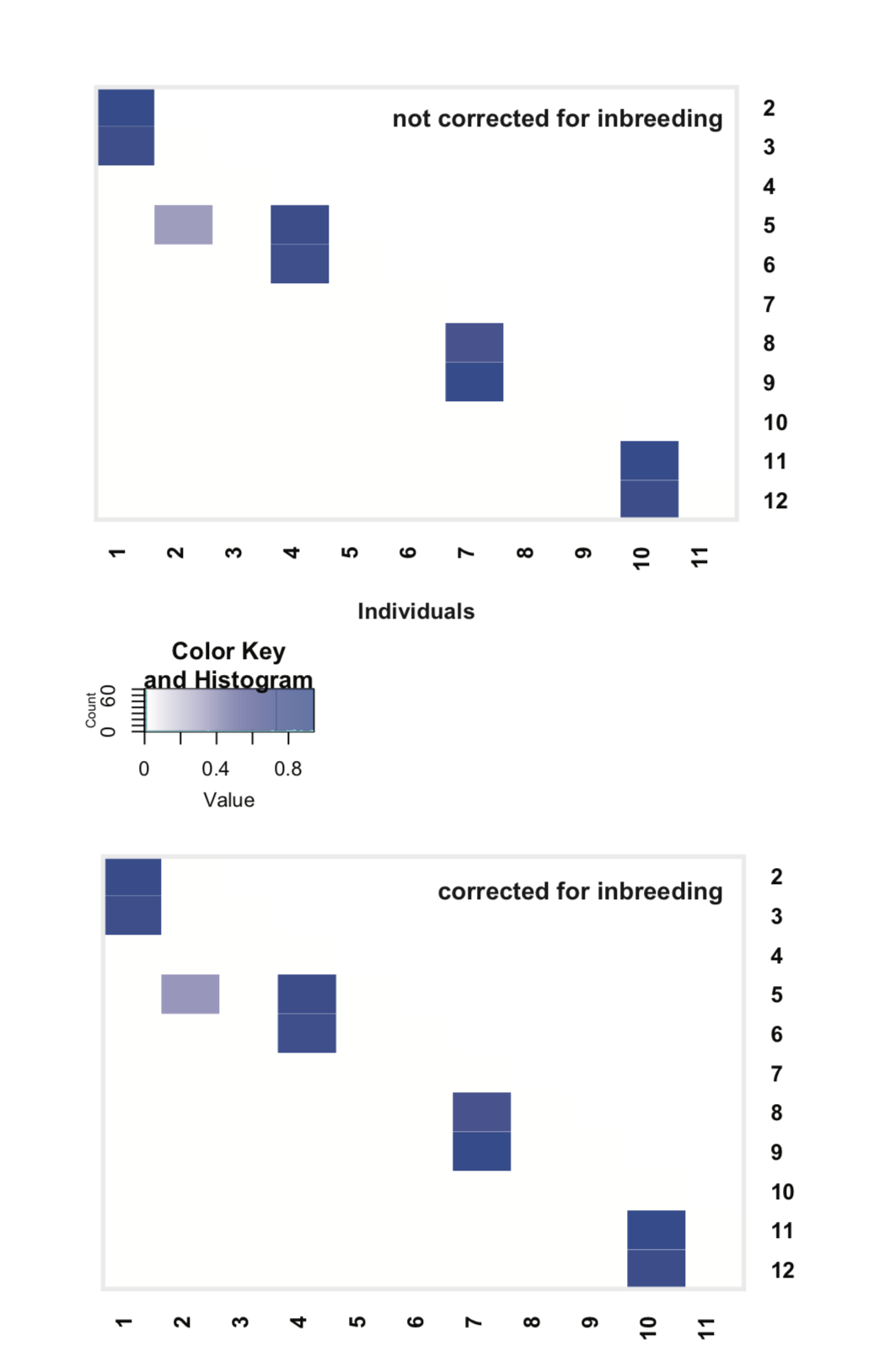


**Supplementary Figure S10: Relatedness (K2) found in the 46 cheetah samples.** Top panel: relatedness inferred without accounting for inbreeding, and bottom panel relatedness inferred after correcting for inbreeding. Darker blue indicates a closer relationship and white color indicates no relationship between individuals. The different subspecies are abbreviated with SOE for *A. j. soemmeringii,* JUB for *A. j. jubatus*, VEN for *A. j. venaticus*, HEC for *A. j. hecki* and RAI for *A. j. raineyi.*


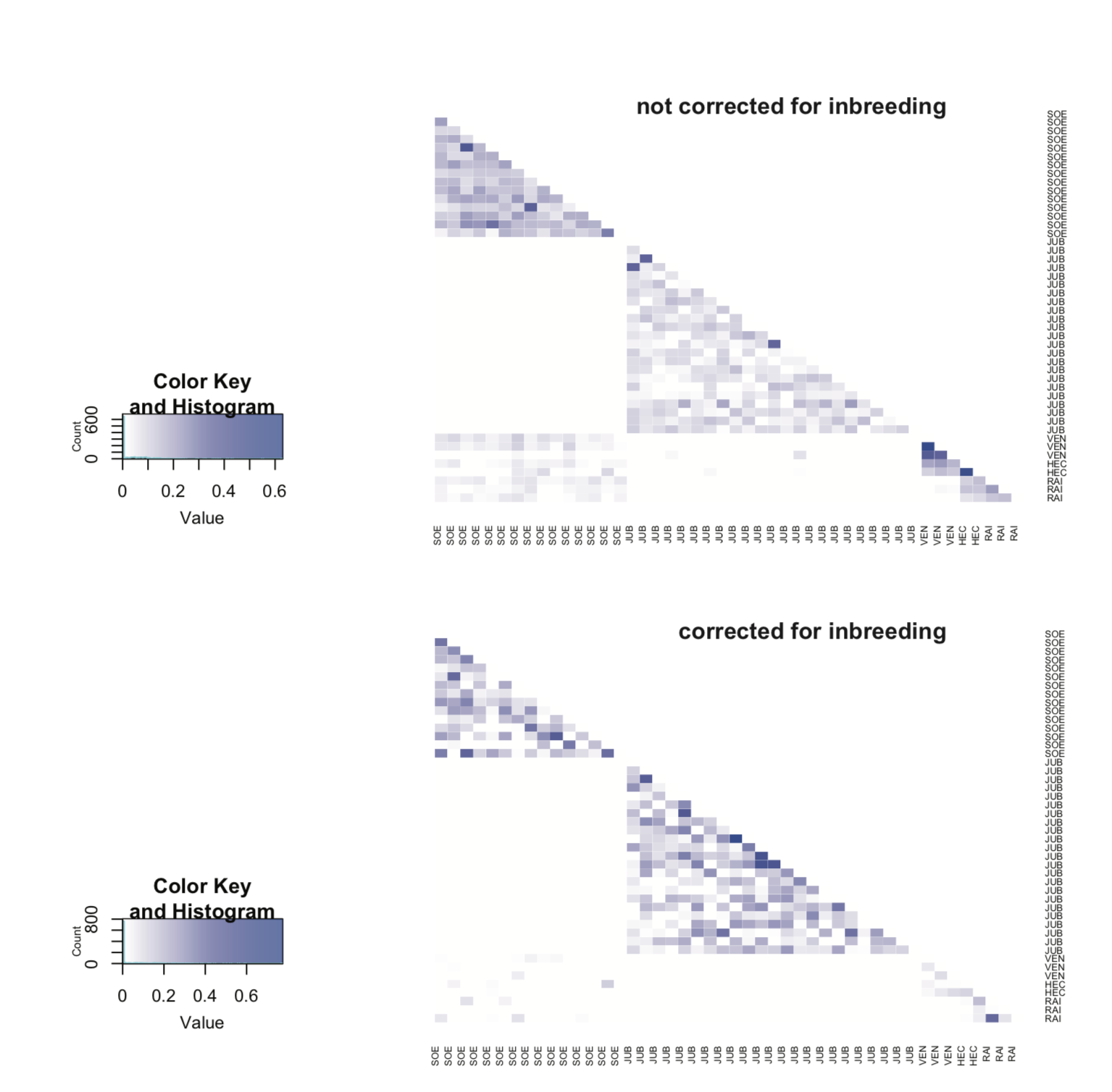


**Supplementary Figure S11: Median-joining haplotype network of 681bp mitochondrial DNA.** Blue: *A. j. venaticus*, green: *A. j. hecki*, grey: *A. j. soemmeringii,* purple: *A. j. jubatus,* or
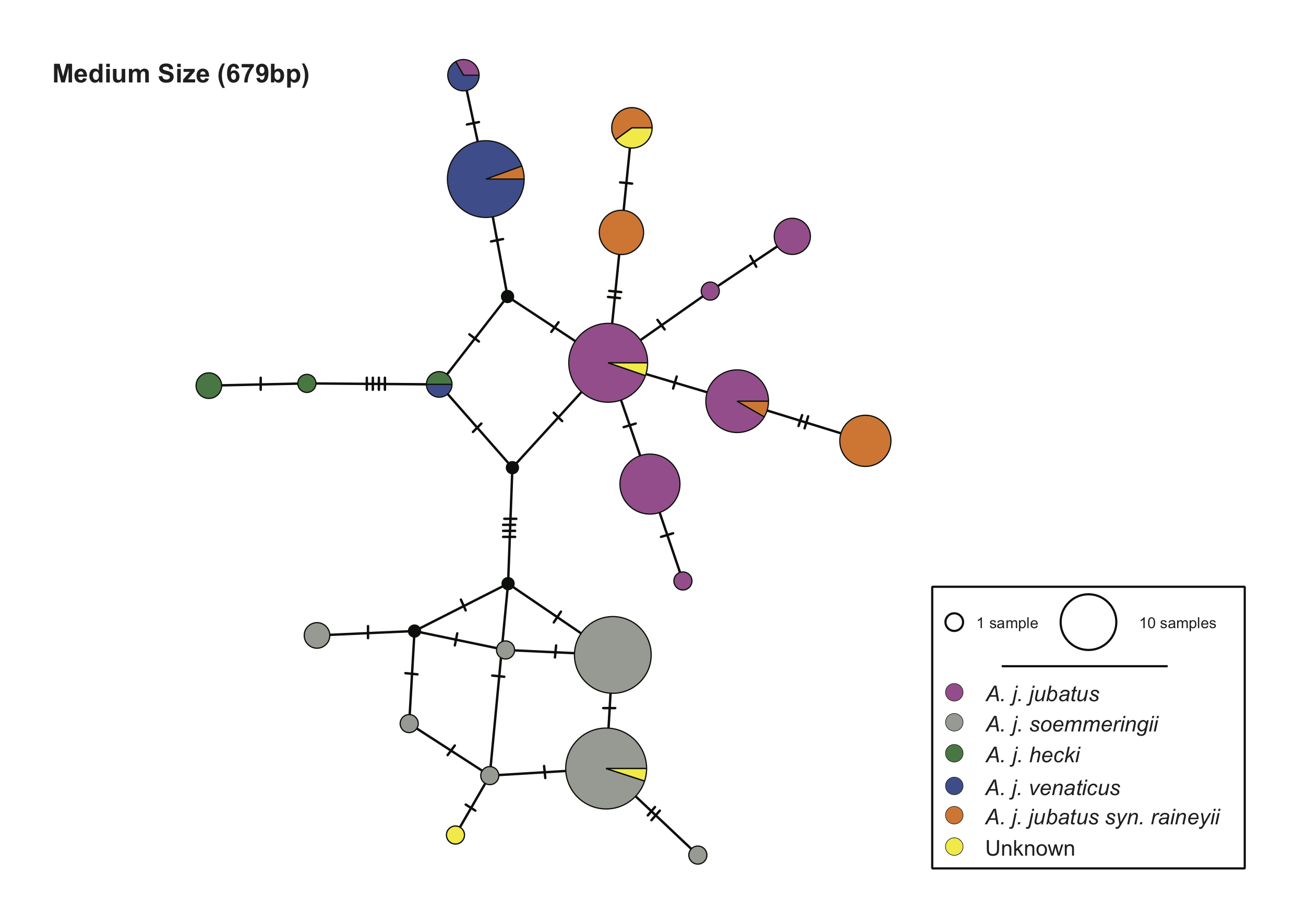
ange: *A. j. raineyi*.

**Supplementary Figure S12: Median-joining haplotype network of 190bp mitochondrial DNA.** Blue: *A. j. venaticus*, green: *A. j. hecki*, grey: *A. j. soemmeringii,* light grey: *A. j. soemmeringii* from the ddRAD analysis*,* purple: *A. j. jubatus,* orange: *A. j. raineyi*. We removed samples with questionable or unknown origin for this analysis.


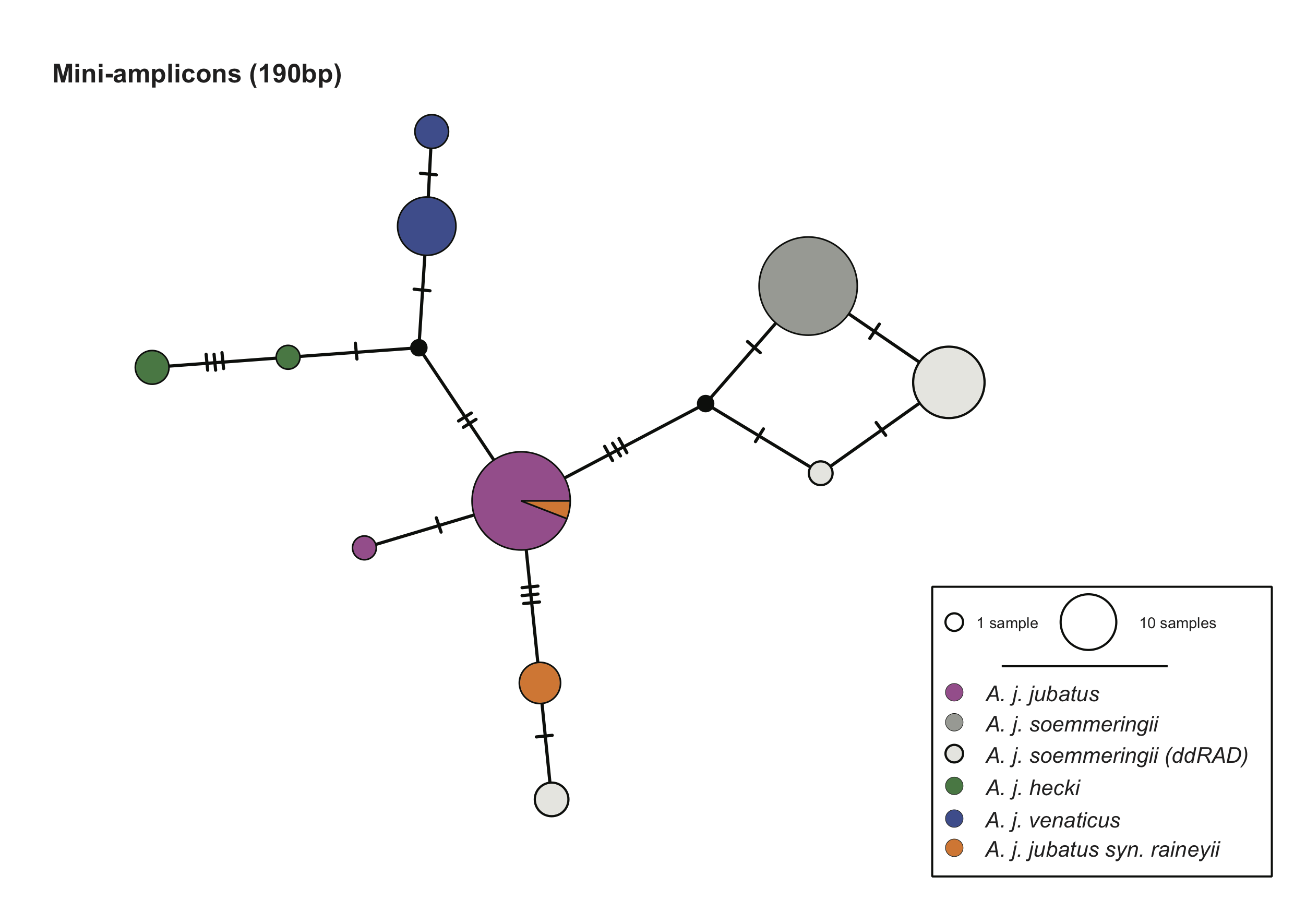


**Supplementary Figure S13: Rarefaction analyses on the number of MHC II DRB exon 2 haplotypes in the three different subspecies: *A. j. jubatus* (purple), *A. j. soemmeringii***
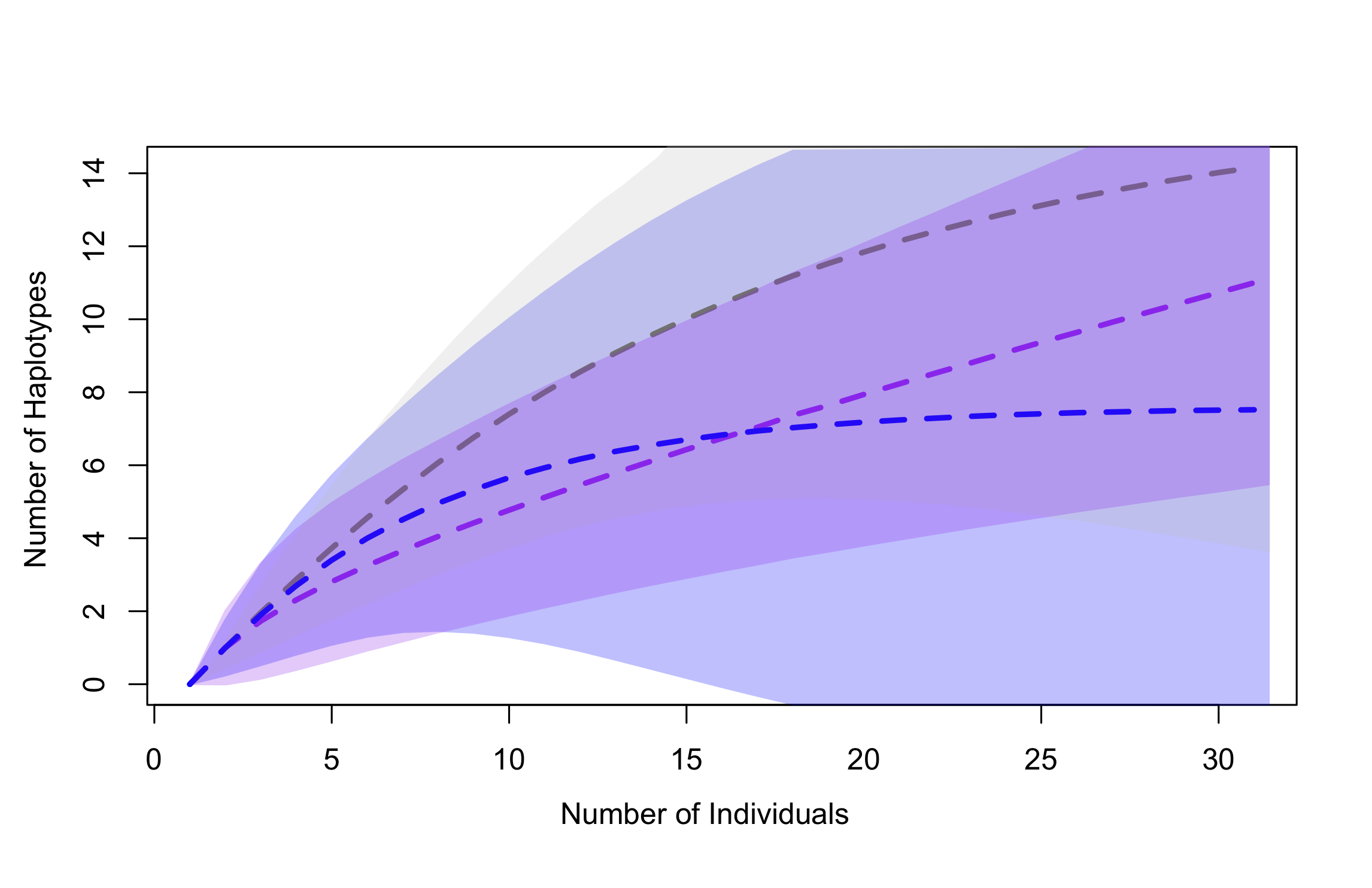
**(grey) and *A. j. venaticus* (blue).**

**Supplementary Figure S14: Admixture results for the run including the two *A. j. soemmeringii* ddRAD samples (*) that showed mitochondrial haplotypes of the *A. j. raineyi* haplogroup.**


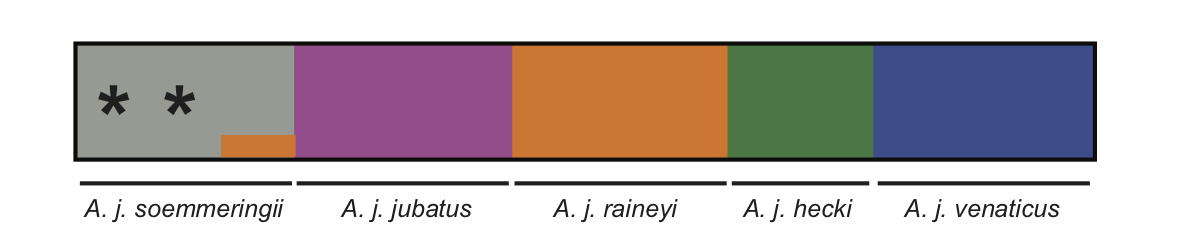


**Supplementary Tables**

**Supplementary Table S1: CITES registered institutions and their registration numbers.** Samples were transported between institutions under the listed CITES registration numbers.

| **Institution** | **CITES Registration Number** |
| --- | --- |
| Forschungsinstitut für Wildtierkunde und Ökologie, Vetmeduni Vienna, Österreich | **AT031** |
| cE3c – Centre for Ecology, Evolution and Environmental Changes, Faculdade de Ciências da Universidade de Lisboa, Portugal | **PT004** |
| Koret School Veterinary Medicine, Hebrew University of Jerusalem, Israel | **IL002** |
| Museo Civico di Storia Naturale Giacomo Doria, Genova, Italy | **IT019** |
| Natural History Museum “La Specola”, Florence, Italy | **IT008** |
| Museum of Evolution, Uppsala, Sweden | **SE010** |
| Zoologisches Museum der Universität Zürich, Switzerland | **CH005** |
| Natural History Museum of Geneva, Switzerland | **CH004** |
| Naturhistorisches Museum Basel, Switzerland | **CH002** |
| Naturmuseum Sankt Gallen, Switzerland | **CH033** |
| Field Museum of Natural History, Chicago, USA | **US012** |
| Harvard University Museum of Comparative Zoology,Cambridge, 02138 | **US051** |
| Natural History Museum, London, England | **GB001** |
| Botswana National Museum, Gaborone, Botswana | **BW004** |
| Tel Aviv University, Israel | **IL001** |
| Leiden University, Institute of Biology Dept Integrative Zoology, Leiden, Netherlands | **NL012** |
| Museum für Naturkunde Berlin, Berlin, Germany | **DE203-06** |
| Real jardín botánico Consejo superior de investigaciones cientificas, Madrid, Spain | **ES001** |
| Museum National d’Histoire Naturelle, Paris, France | **FR75A** |
| Natural History Museum Denmark, Copenhagen, Denmark | **DK003** |
| Kwa-Zulu Natal Museum, Pietermaritzburg, South Africa | **ZA025** |
| Amathole Museum, King William’s Town, South Africa | **ZA020** |
| National Zoological Gardens of South Africa, Pretoria, South Africa | **ZA034** |

**Supplementary Table S3: *F*_ST_ values between the five classical subspecies of cheetahs based on the three downsampled replicate sets.**

|  | ***A. j. hecki*** | ***A. j. raineyi*** | ***A. j. soemmeringii*** | ***A. j. venaticus*** | ***A. j. jubatus*** |
| --- | --- | --- | --- | --- | --- |
| ***A. j. jubatus set 1*** | 0.392755 | 0.247042 | 0.223748 | 0.47101 | - |
| ***A. j. jubatus set 2*** | 0.392755 | 0.247039 | 0.22375 | 0.47101 | - |
| ***A. j. jubatus set 3*** | 0.392753 | 0.247042 | 0.223751 | 0.471009 | - |
| ***A. j. soemmeringii set 1*** | 0.396215 | 0.218642 | - | 0.438073 | 0.285415 |
| ***A. j. soemmeringii set 2*** | 0.384048 | 0.243924 | - | 0.440956 | 0.295766 |
| ***A. j. soemmeringii set 3*** | 0.369395 | 0.200246 | - | 0.417954 | 0.263241 |

**Supplementary Table S4: *F*_ST_ values between the five classical subspecies of cheetahs based on the full data set of 46 individuals.**

| **FST** | ***A. j. jubatus*** **(n=23)** | ***A. j. venaticus*** **(n=3)** | ***A. j. hecki*** **(n=2)** | ***A. j. raineyi*** **(n=3)** |
| --- | --- | --- | --- | --- |
| ***A. soemmeringii*** **(n=15)** | 0.193 | 0.360 | 0.399 | 0.157 |
| ***A. j. jubatus*** **(n=23)** |  | 0.375 | 0.410 | 0.192 |
| ***A. j. venaticus*** **(n=3)** |  |  | 0.497 | 0.475 |
| ***A. j. hecki*** **(n=2)** |  |  |  | 0.369 |

**Supplementary Table S5: Characteristics of the primers employed in this study.** aMT indicates the primers used for highly fragmented museum samples.

| **Gene** | **Amplicon** | **Ta (°C)** | **Size (bp) including primer sequences** | **Primer** **(forward)** | **Primer sequence** | **Primer** **(reverse)** | **Primer sequence** |
| --- | --- | --- | --- | --- | --- | --- | --- |
| **ND5** | ND5.1 | 52 | 310 | tSER-12564f | CGTATAAAAACGTGGCTTT | ND5int-12875r | GGTTTGGATTGATAATCAGTGT |
|  | ND5.2 | 48 | 375 | ND5int-12766f | AAACCACAATCTCATACGC | ND5-13140r | TTCGGCCATATCATCAC |
|  | aMT-ND5 | 59 | 53 | aMT - ND5F | TGGTGCAACTCCAAATAAAAGTA | aMT – ND5R | AATCGGGTGTTAGATATGATGATG |
| **CR** | CR1 | 57 | 250 | CR 16252f | TCACACCCTCCCTAAGACTTCA | CR 16503r | ACGGGGTGGTTGATAGATTAAT |
|  | CR3 | 57 | 245 | CR 16787f | TAAGACATTACAGTGTTTGGTCGT | CR 17029 | AGAAAGTTGAAGGATTGGATTC |
|  | aMT-CR1 | 59 | 41 | aMT - CR1F | TCCAACAAAACAAACCAAGTAAA | aMT - CR1R | GACGGGGTGGTTGATAGATT |
|  | aMT-CR3 | 58 | 45 | aMT - CR3F | TAAGACATTACAGTGTTTGGTCGT | aMT - CR3R | GCTAGGTGATTTAGGTTCCTTTG |
| **Mini-barcodes** | Amp1 | 56 | 130 | Amp1f | TGCAACTCCAAATAAAAGTAATAA | Amp1r | TTTACATAGTGGGGGTATAAGTTG |
|  | Amp4 | 60 | 90 | Amp4f | CACTTCCAACAAAACAAACCAA | Amp4r | GAGACGGGGTGGTTGATAGA |
|  | Amp5 | 60 | 101 | Amp5f | CGGGACAATTCTCTATGGA | Amp5r | GGAGCGAGAAGAGGTACAC |
| **MHC class II DRB exon 2** |  | 62 | 237 | DRB_SL-F | GCGTCAGTGTCTTCCAGGAG | DRB_SL-R | GGGACCCAGTCTCTGTCTCA |
